# Supplementary material for: GECSI: large-scale chromatin state imputation from gene expression
Source: Genome Biol. 2026 Aug 1;27:329. doi: 10.1186/s13059-026-04209-5 (PMC13721437; doi:10.1186/s13059-026-04209-5)
Supplement: Supplementary file 1 [file 13059_2026_4209_MOESM2_ESM.pdf]

Supplementary Figures

GECSI: Large-scale chromatin state imputation from gene expression

Jingyuan Fu, Jason Ernst

Contents

Supplementary Figures 1-30

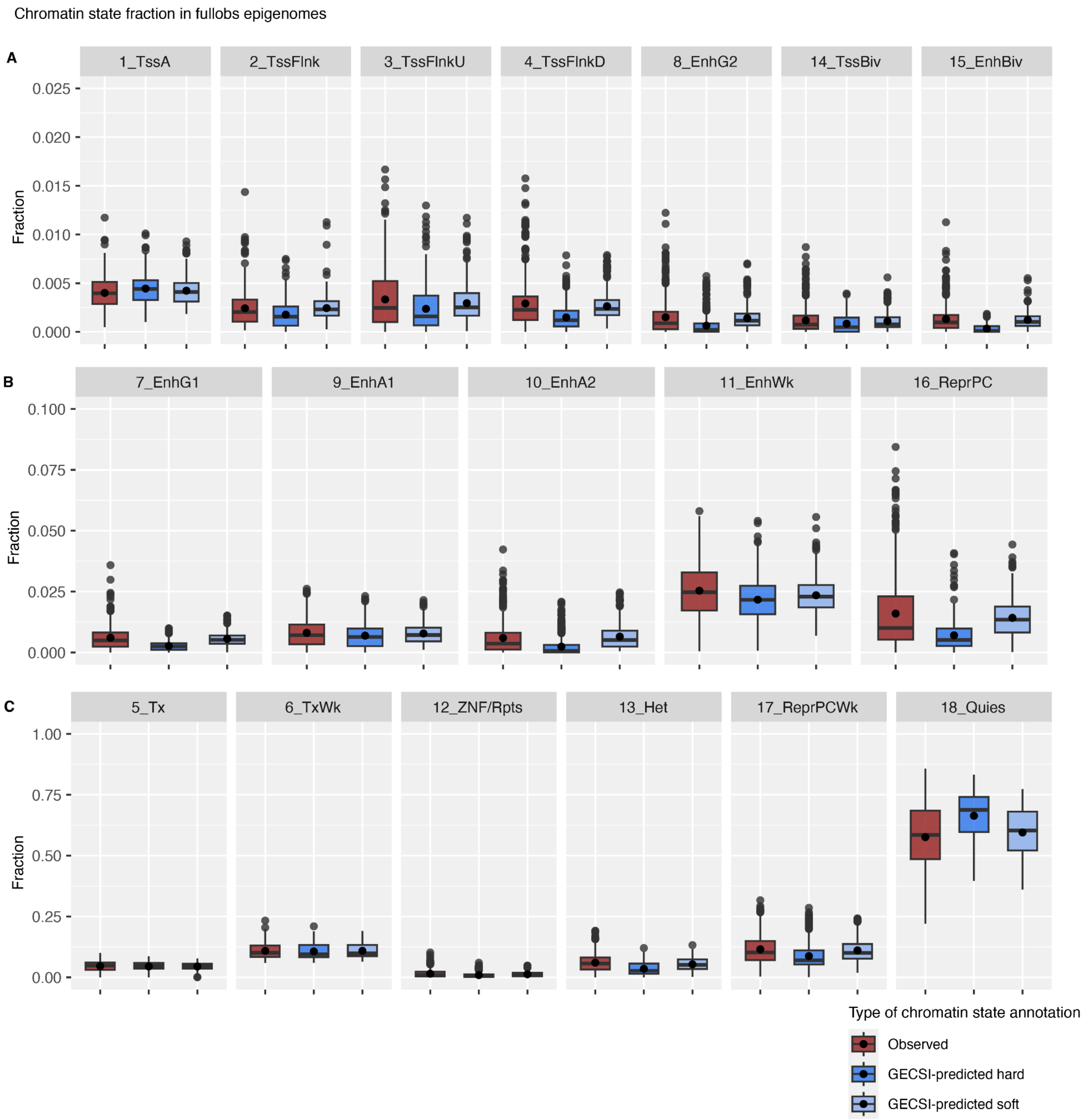

Fig. S1: Chromatin state genome coverage fractions for fully observed (fullobs) epigenomes. Each plot displays for one of the 18 chromatin states the distribution of the genome fraction of the state across all 414 fullobs reference epigenomes with separate boxplots based on observed chromatin state annotations (red), GECSI-predicted chromatin state hard assignments (blue), and GECSI-predicted chromatin state soft assignments (light blue). The plots are grouped based on the size of the chromatin states, with each sub-panel sharing one y-axis scale: **(A)** 0-0.025, **(B)** 0-0.1, and **(C)** 0-1. GECSI predictions were made in cross-validation. For each box, the interquartile range (IQR) is from the 25th to 75th percentile, with the horizontal line inside the box indicating the median, and the dot inside the box indicating the mean. Whiskers extend to the smallest and largest values within 1.5 × IQR from the lower and upper quartiles, respectively; points beyond this range are shown as outliers. Descriptions that correspond to the chromatin state abbreviations are available in **Additional File 1: Table S1**.

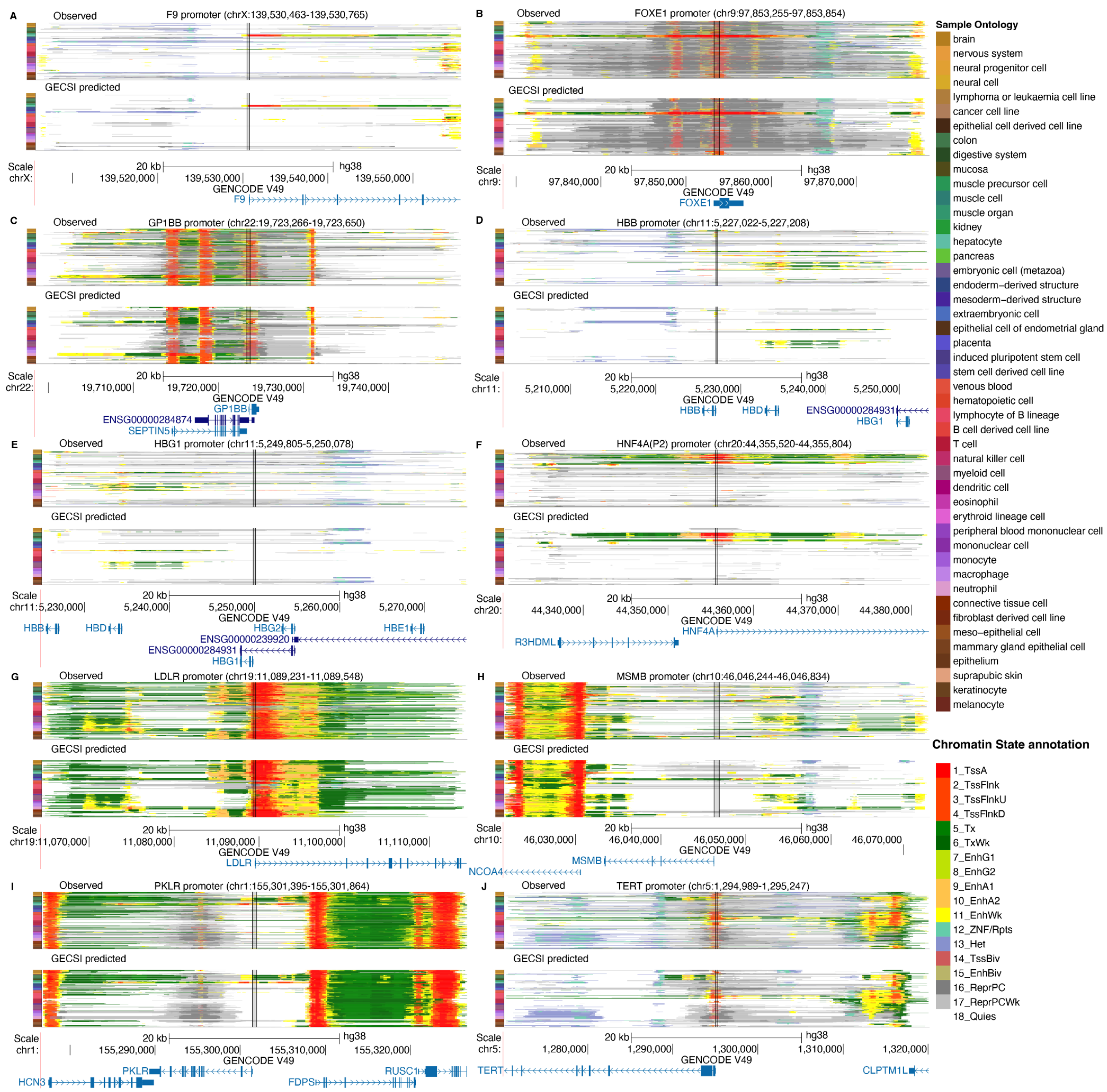

Fig. S2: Visualizations of GECSI-predicted and observed chromatin state annotations in selected disease-associated gene promoter regions. Visualization of chromatin state tracks of 414 fully observed IHEC reference epigenomes based on observed data (top) and GECSI's predictions (bottom) for 50kb regions around ten previously curated disease-associated gene promoters (Kircher et al., 2019): **(A)** F9 (chrX:139505614-139555614), **(B)** FOXE1 (chr9:97828554-97878554), **(C)** GP1BB (chr22:19698458-19748458), **(D)** HBB (chr11:5202115-5252115), **(E)** HBG1 (chr11:5224941-5274941), **(F)** HNF4A(P2) (chr20:44330662-44380662), **(G)** LDLR (chr6:105452601-105652600), **(H)** MSMB (chr10:46021539-46071539), **(I)** PKLR (chr1:155276629-155326629), **(J)** TERT (chr5:1270118-1320118). Each row is a chromatin state track for an IHEC reference epigenome. Genomic coordinates of the corresponding promoters are highlighted by black lines and labelled on top of each plot. Tracks are ordered by a previously defined ordering by IHEC. The color label on the left is based on intermediate sample ontology groups from the IHEC metadata. Annotations generated by UCSC genome browser with GENCODE v48 and Genome assembly GRCh38. Chromatin state annotations follow the color coding as previously (Roadmap Epigenomics Consortium et al., 2015) and the color coding is shown in the lower right corner, with detailed descriptions of the annotations provided in **Additional File 1: Table S1**.

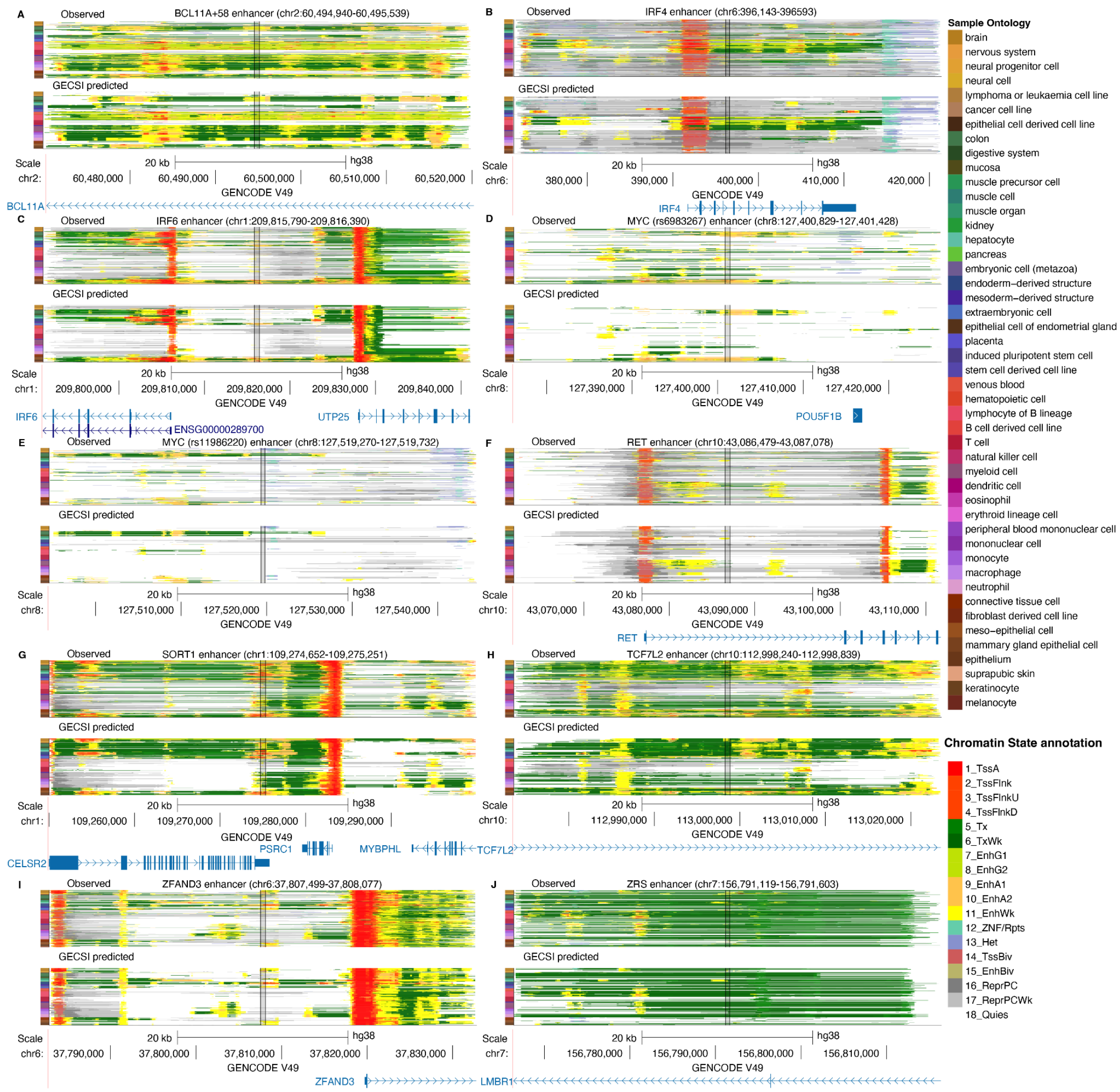

Fig. S3: Visualizations of GECSI-predicted and observed chromatin state annotations in selected disease-associated gene enhancer regions. Visualization of chromatin state tracks of 414 fully observed IHEC reference epigenomes based on observed data (top) and GECSI's predictions (bottom) for 50kb regions around ten previously curated disease-associated gene enhancers (Kircher et al., 2019): **(A)** BCL11A+58 (chr2:60470240-60520240), **(B)** IRF4 (chr6:371368-421368), **(C)** IRF6 (chr1:209791090-209841090), **(D)** MYC (rs6983267) (chr8:127376128-127426128), **(E)** MYC (rs11986220) (chr8:127494501-127544501), **(F)** RET (chr10:43061778-43111778), **(G)** SORT1 (chr1:109249952-109299952), **(H)** TCF7L2 (chr10:112973540-113023540), **(I)** ZFAND3 (chr6:37782788-37832788), **(J)** ZRS (chr7:156766361-156816361). Each row is a chromatin state track for an IHEC reference epigenome. Genomic coordinates of the corresponding enhancers are highlighted by black lines and labelled on top of each plot. Tracks are ordered by a previously defined ordering by IHEC. The color label on the left is based on intermediate sample ontology groups from the IHEC metadata. Annotations generated by UCSC genome browser with GENCODE v48 and Genome assembly GRCh38. Chromatin state annotations follow the color coding as previously (Roadmap Epigenomics Consortium et al., 2015) and the color coding is shown in the lower right corner, with detailed descriptions of the annotations provided in **Additional File 1: Table S1**.

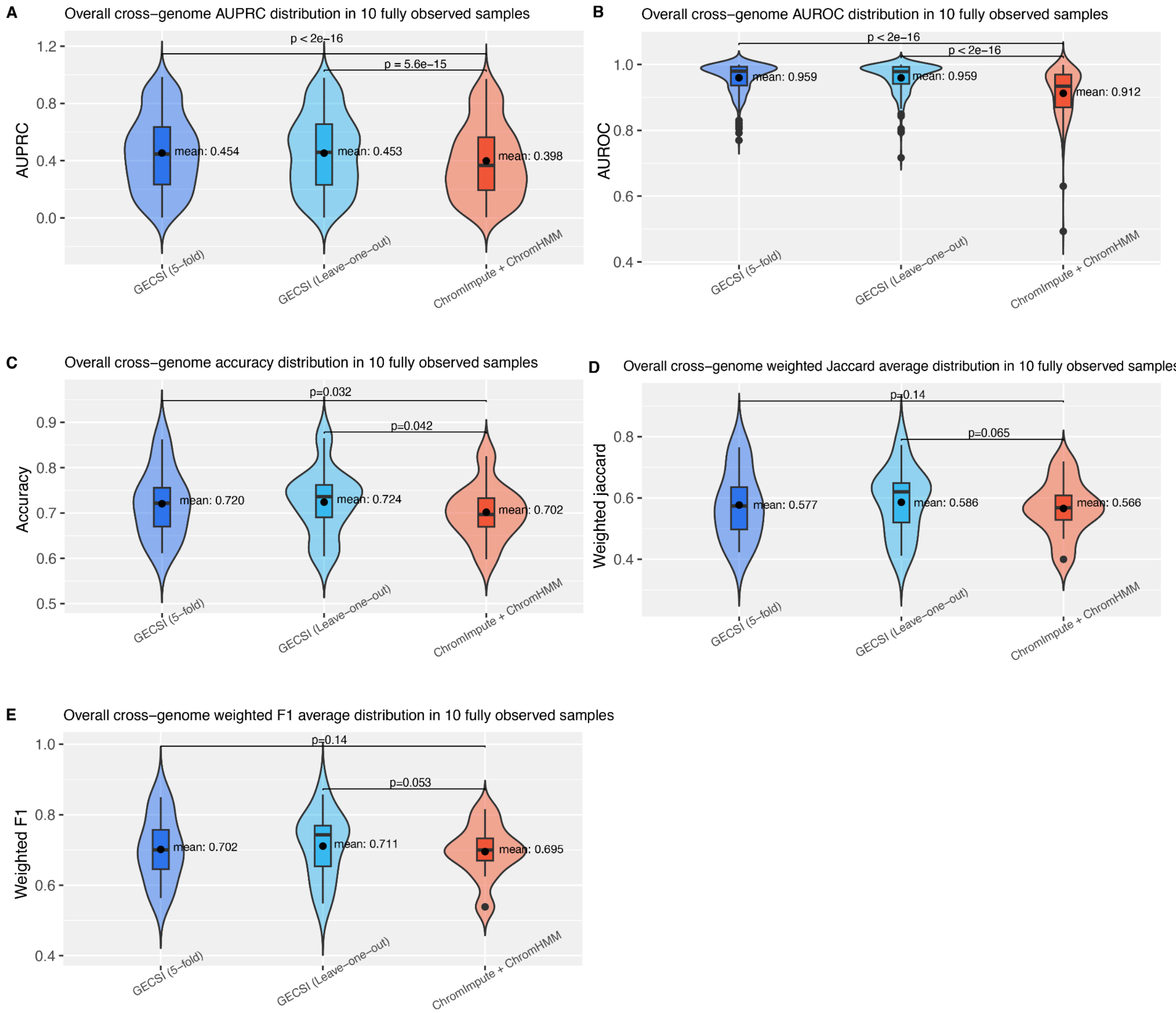

Fig. S4: Evaluations of chromatin state predictions in different experimental settings in 10 fullobs epigenomes. Shown are the results for soft and hard assignments predicting 18 chromatin states among 10 randomly selected fullobs reference epigenomes. The results are obtained from GECSI using a 5-fold cross validation setting, GECSI using a leave-one-out setting, and ChromImpute+ChromHMM using a leave-one-out setting, respectively. Specifically, violin and boxplots showing the distribution across all combinations of chromatin states and the 10 epigenomes for **(A)** AUPRC, **(B)** AUROC, **(C)** accuracy, **(D)** weighted Jaccard averages, and **(E)** weighted F1 averages.

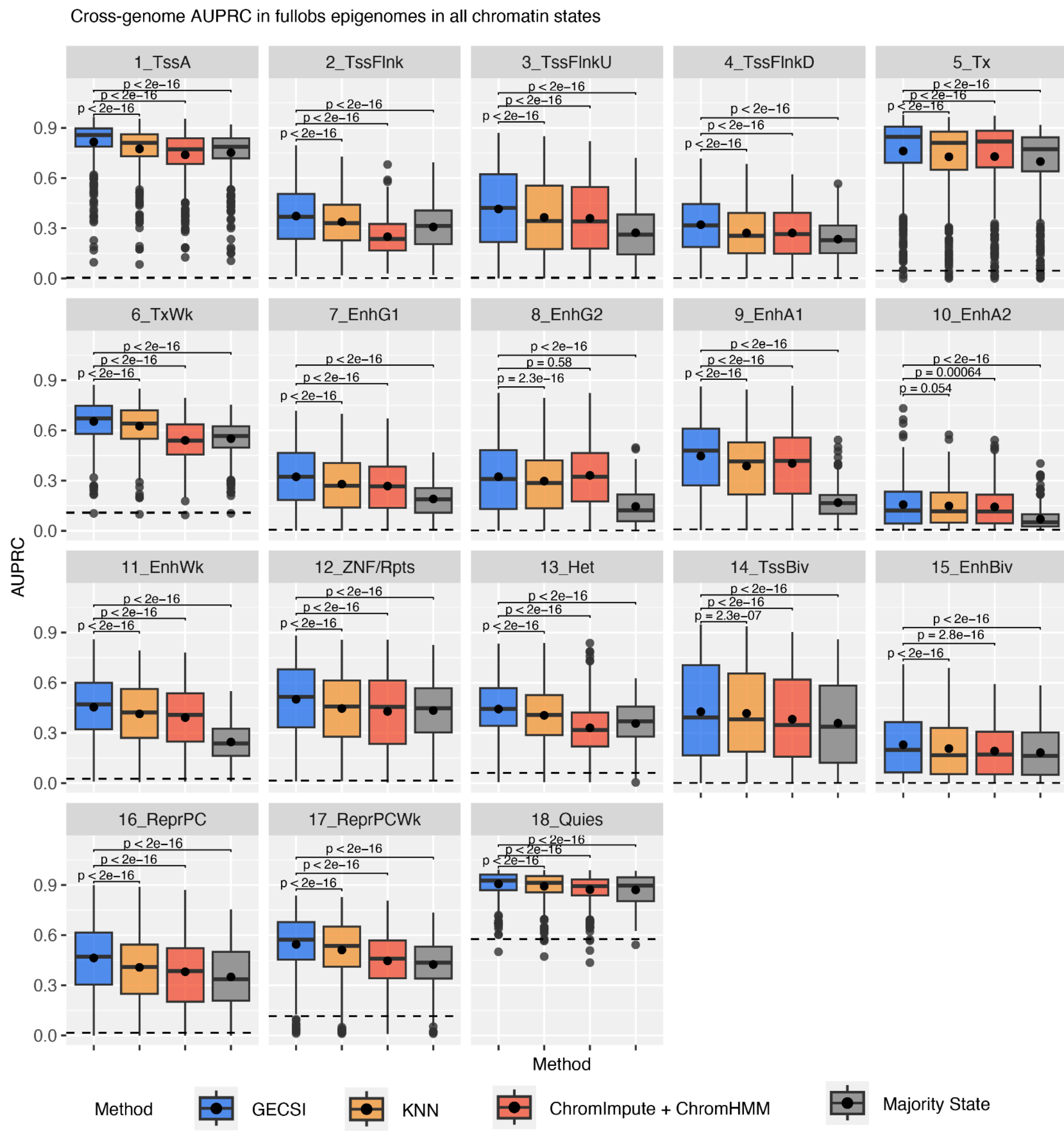

Fig. S5: Evaluations on AUPRC scores in the fully observed (fullobs) epigenomes. Boxplots showing cross-validation results for AUPRC based on soft assignments predicting 18 chromatin states among 414 fullobs reference epigenomes. Each plot displays the AUPRC distribution across all fullobs reference epigenomes for one of the chromatin states. Each box corresponds to one of the methods evaluated: GECSI, KNN, ChromImpute+ChromHMM, or Majority State. Statistical significance between GECSI and other methods was assessed using a one-sided Wilcoxon signed-rank test. For each box, the interquartile range (IQR) is from the 25th to 75th percentile, with the horizontal line inside the box indicating the median, and the dot inside the box indicating the mean. Whiskers extend to the smallest and largest values within 1.5 × IQR from the lower and upper quartiles, respectively; points beyond this range are shown as outliers. Black dashed lines in each plot represents expected AUPRC by random guessing given the positive/negative class ratios.

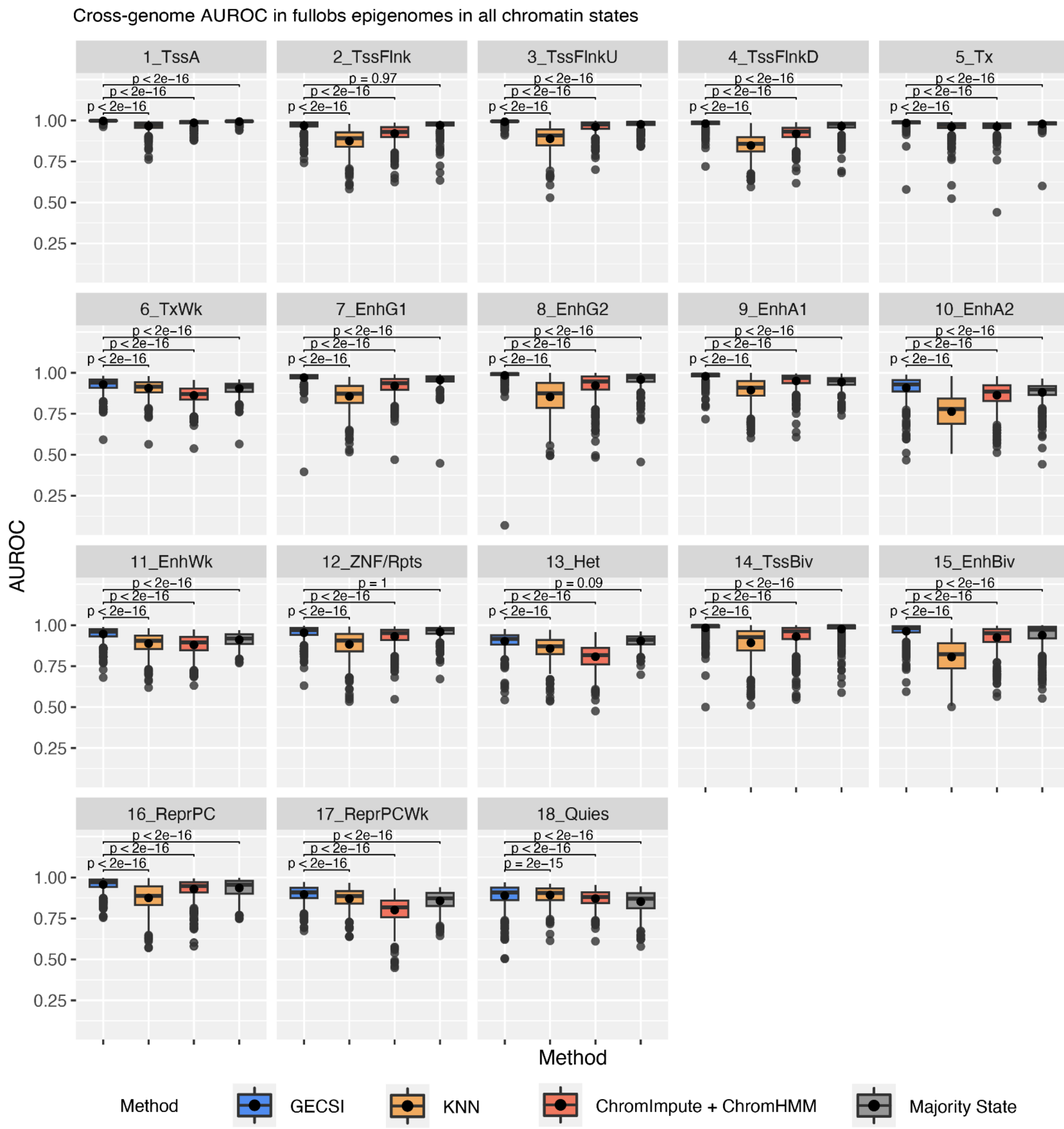

Fig. S6: Evaluations on AUROC scores in the fullobs epigenomes. Boxplots showing cross-validation results for AUROC based on soft assignments predicting 18 chromatin states among 414 fullobs reference epigenomes. Each plot displays the AUROC distribution across all fullobs reference epigenomes for one of the chromatin states. Each box corresponds to one of the methods evaluated: GECSI, KNN, ChromImpute+ChromHMM, and Majority State. Statistical significance between GECSI and other methods was assessed using a one-sided Wilcoxon signed-rank test. For each box, the interquartile range (IQR) is from the 25th to 75th percentile, with the horizontal line inside the box indicating the median, and the dot inside the box indicating the mean. Whiskers extend to the smallest and largest values within 1.5 × IQR from the lower and upper quartiles, respectively; points beyond this range are shown as outliers.

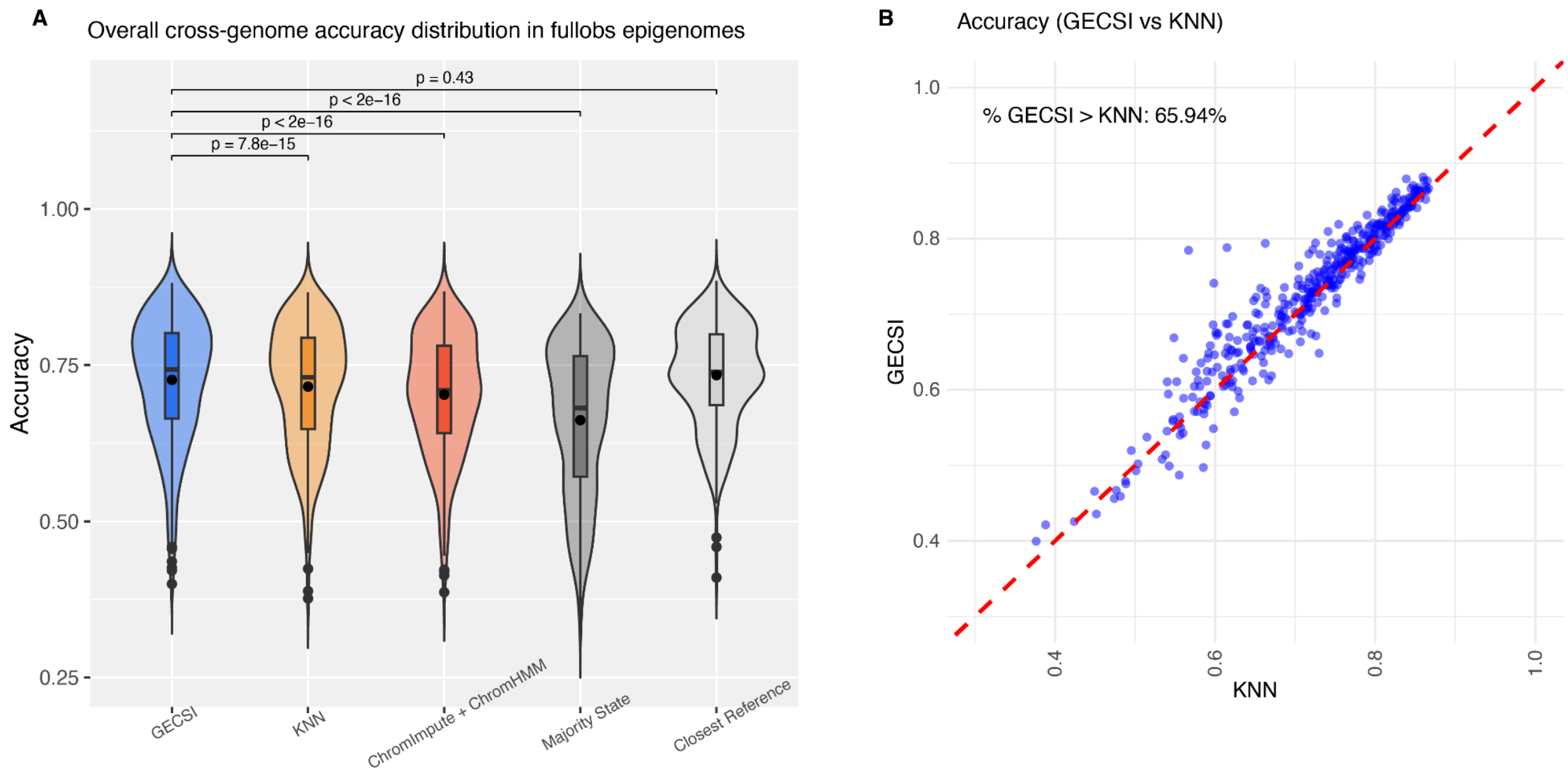

Fig. S7: Evaluations on chromatin state prediction accuracy in the fullobs epigenomes. Shown are cross-validation results for accuracy of predicted hard chromatin state assignments among 414 fullobs reference epigenomes, specifically: **(A)** Violin and boxplots showing the distribution of accuracy across all combinations of epigenomes and chromatin states separately for GECSI, KNN, ChromImpute+ChromHMM, Majority State, and the closest reference benchmark. Statistical significance between GECSI and other methods was assessed using a one-sided Wilcoxon signed-rank test. **(B)** Scatterplot comparing accuracy of GECSI method (y-axis) and KNN (x-axis), with the red dashed diagonal line representing y=x. The percentage of points with higher accuracy based on GECSI compared to KNN is shown in the upper left corner of the plot.

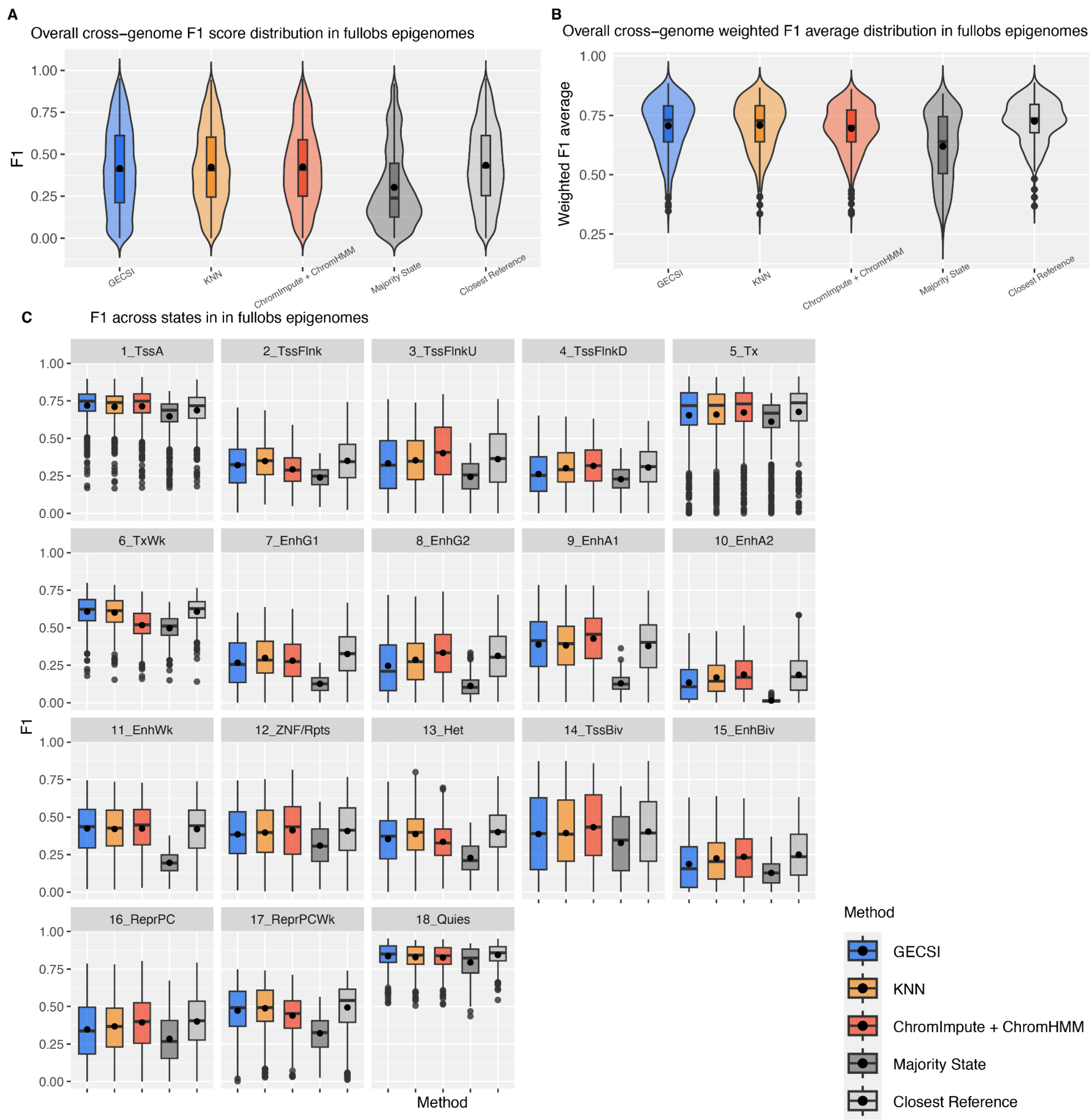

Fig. S8: Evaluations on F1 scores and weighted F1 averages in the fullobs epigenomes. Shown are cross-validation results for F1 score and weighted F1 metrics based on predicted hard chromatin state assignments for 18 chromatin states among 414 fullobs reference epigenomes, comparing GECSI, KNN, ChromImpute+ChromHMM, Majority State, and the closest reference benchmark, specifically: **(A)** Violin and boxplots showing the distribution of F1 scores across all combinations of epigenomes and chromatin states. **(B)** Violin and boxplots showing the distribution of weighted F1 averages across all combinations of epigenomes and chromatin states. **(C)** Boxplots showing the distribution of F1 scores in 18 chromatin states respectively. Each plot displays the corresponding distributions in one chromatin state. For all boxplots, each box represents the interquartile range (IQR) from the 25th to 75th percentile, with the horizontal line inside the box indicating the median, and the dot inside the box indicating the mean. Whiskers extend to the smallest and largest values within 1.5 × IQR from the lower and upper quartiles, respectively; points beyond this range are shown as outliers.

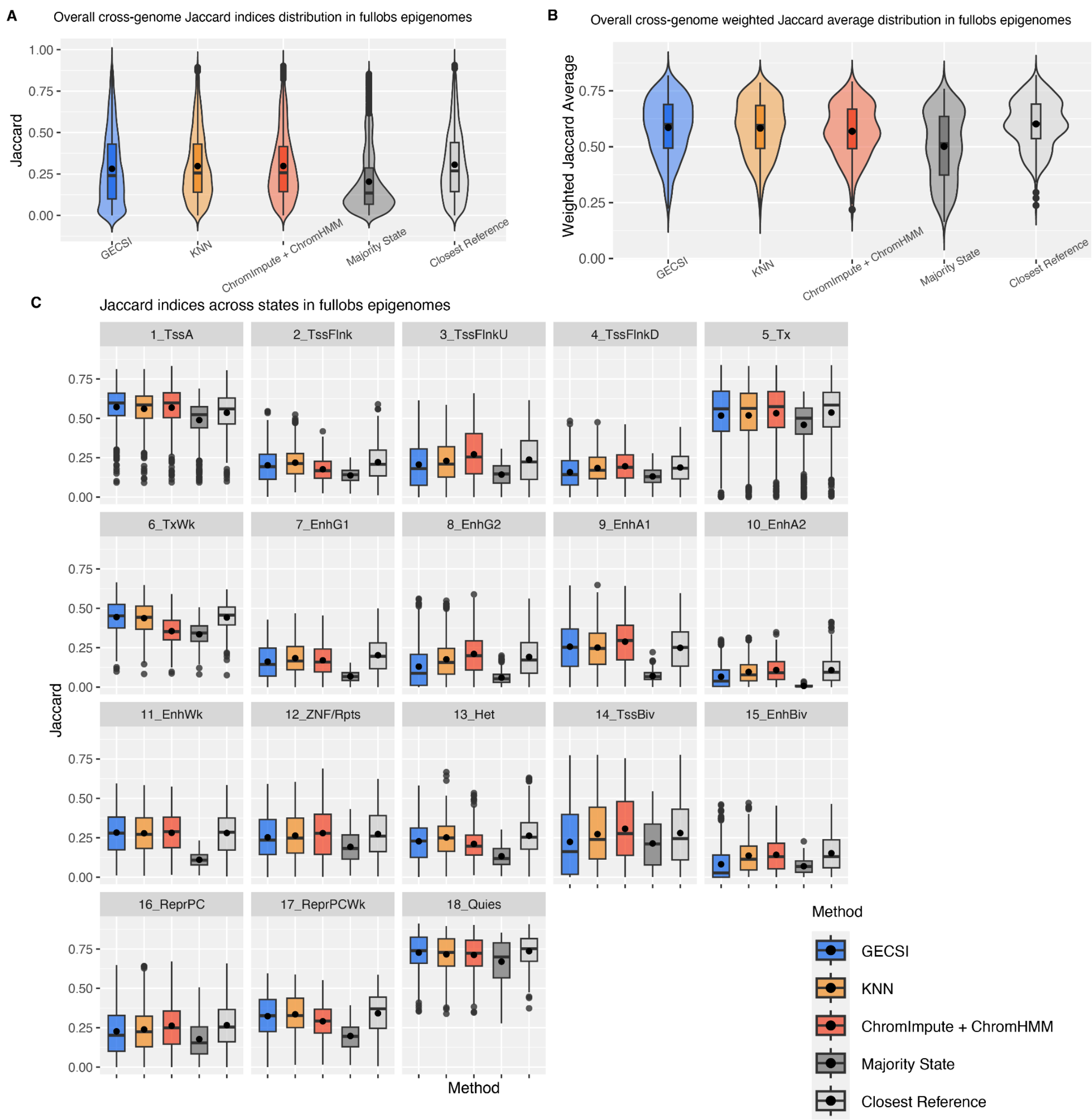

Fig. S9: Evaluations on Jaccard indices and weighted Jaccard averages in the fullobs epigenomes. Shown are cross-validation results for Jaccard index and weighted Jaccard metrics based on predicted hard assignments predictions for 18 chromatin states among 414 fullobs reference epigenomes, comparing GECSI, KNN, ChromImpute+ChromHMM, Majority State, and the closest reference benchmark, specifically: **(A)** Violin and boxplots showing the distribution of Jaccard indices across all combinations of epigenomes and chromatin states. **(B)** Violin and boxplots showing the distribution of weighted Jaccard averages across all combinations of epigenomes and chromatin states. **(C)** Boxplots showing the distribution of Jaccard indices in 18 chromatin states respectively. Each plot displays the corresponding distributions in one chromatin state. For all boxplots, each box represents the interquartile range (IQR) from the 25th to 75th percentile, with the horizontal line inside the box indicating the median, and the dot inside the box indicating the mean. Whiskers extend to the smallest and largest values within 1.5 × IQR from the lower and upper quartiles, respectively; points beyond this range are shown as outliers.

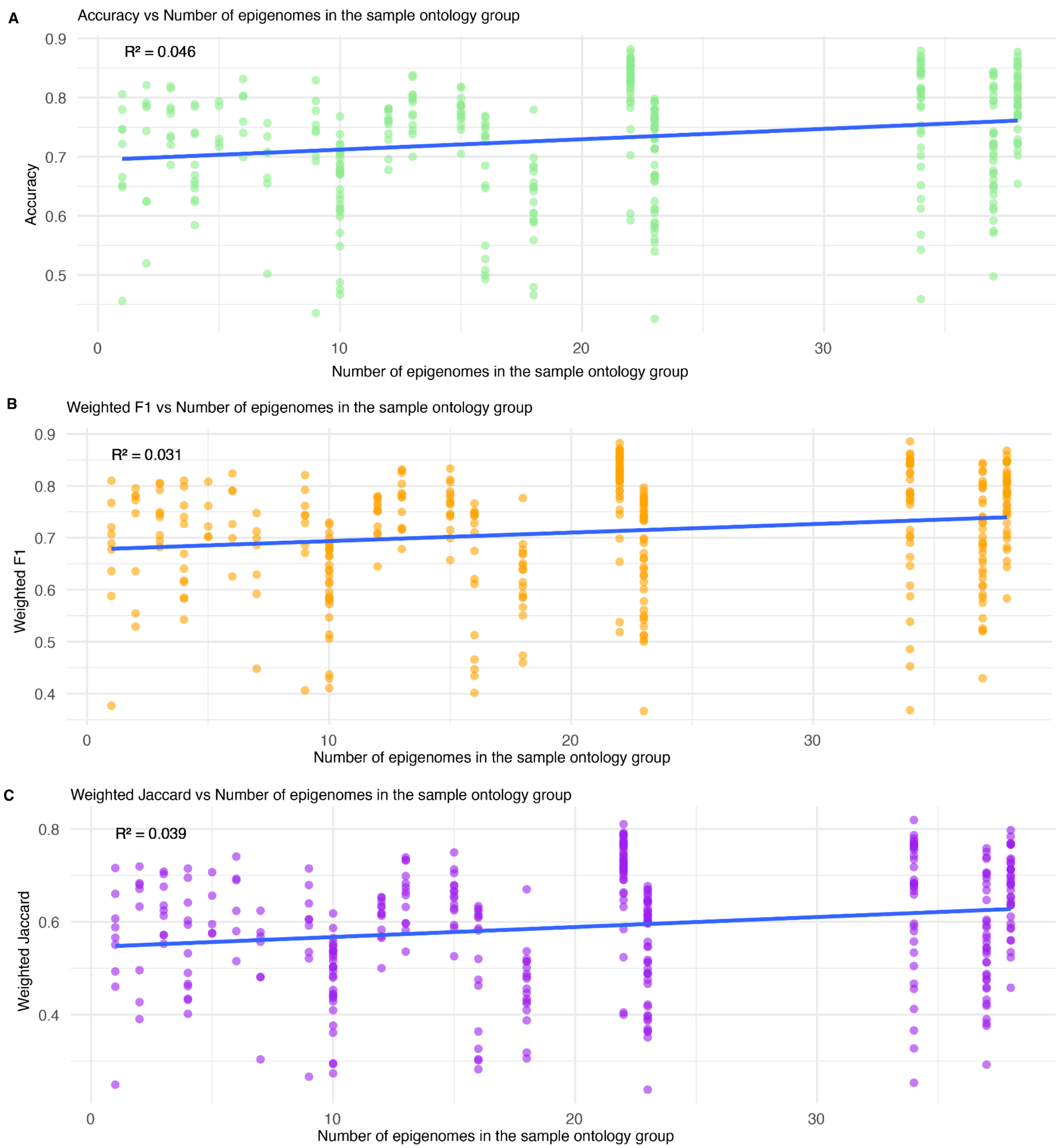

Fig. S10: Evaluation of GECSI's hard assignment predictions agreement with observed assignments as a function of number of epigenomes in the sample ontology group. Scatterplots showing cross-validation results for **(A)** accuracy, **(B)** weighted F1 average, and **(C)** weighted Jaccard average (y-axis) with respect to the number of epigenomes in the compendium that belong to the same sample ontology group as the evaluated epigenome (x-axis). The evaluations are calculated using GECSI-predicted hard chromatin state assignments for 18 chromatin states among 414 fullobs reference epigenomes. The blue line is the line of best fit of the scatterplot, with the coefficient of determination (R^2) labeled on the top left of each plot.

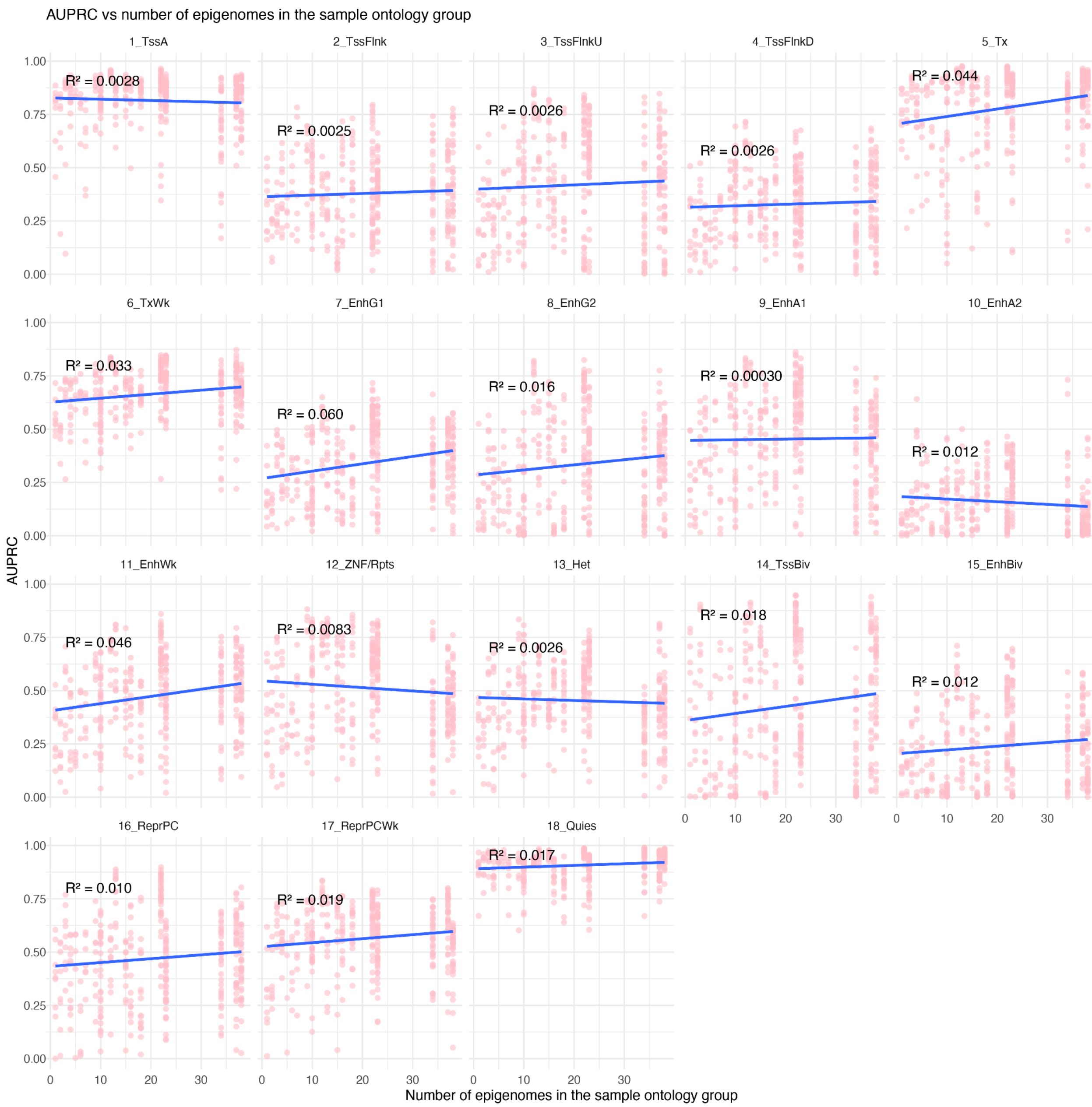

Fig. S11: Evaluation of GECSI's soft assignment predictions' AUPRC for predicting observed assignments as a function of number of epigenomes in the sample ontology group. Scatterplots showing cross-validation results for AUPRC (y-axis) with respect to the number of epigenomes in the compendium that belong to the same sample ontology group as the evaluated epigenome (x-axis). The evaluations are calculated using GECSI-predicted soft chromatin state assignments for 18 chromatin states among 414 fullobs reference epigenomes. Each scatterplot displays the AUPRC scores across all fullobs reference epigenomes for one of the chromatin states, where the blue line is the line of best fit of the scatterplot, with the coefficient of determination (R^2) labeled on each plot.

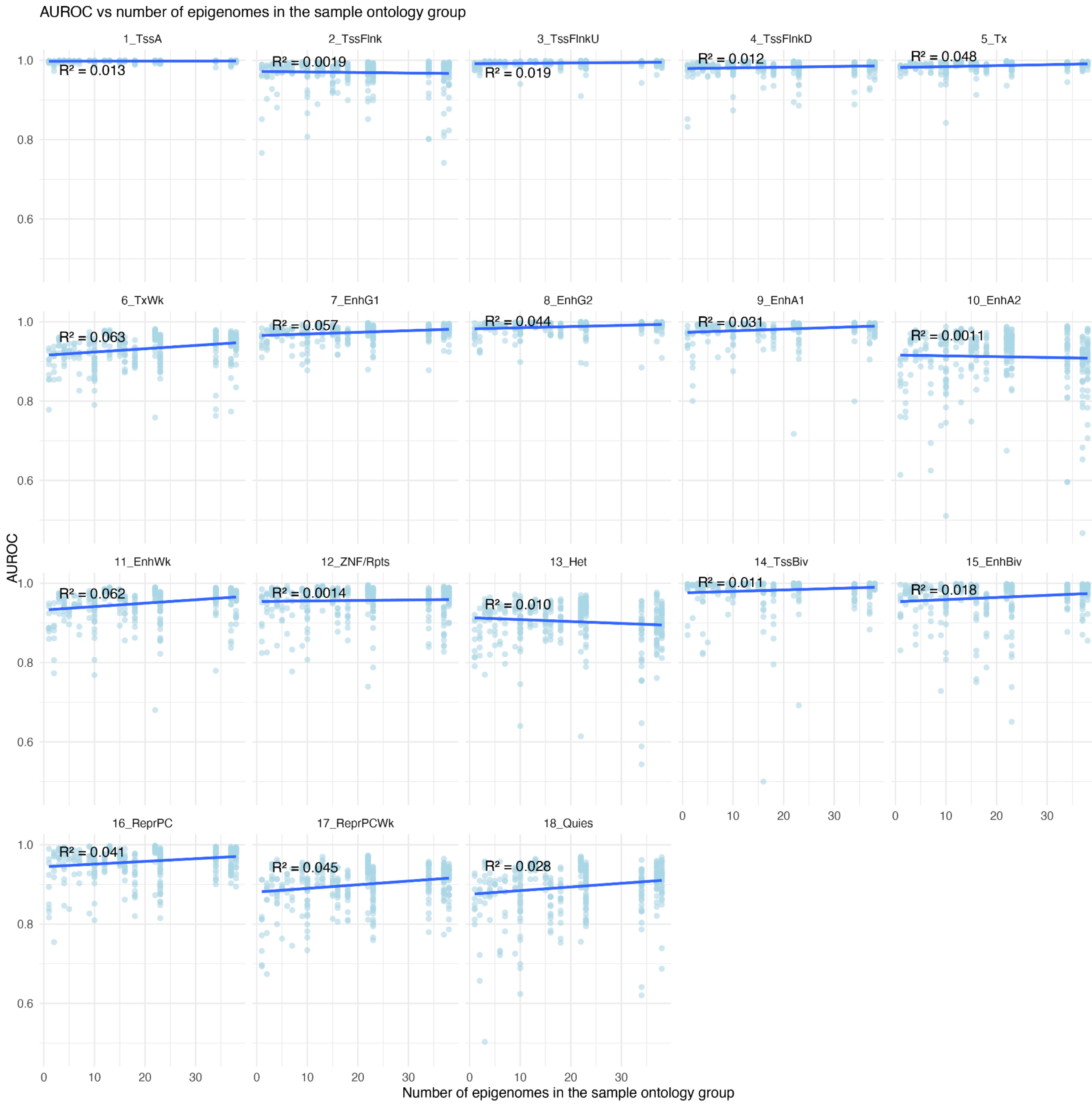

Fig. S12: Evaluation of GECSI's soft assignment predictions' AUROC for predicting observed assignments as a function of number of epigenomes in the sample ontology group. Scatterplots showing cross-validation results for AUROC (y-axis) with respect to the number of epigenomes in the compendium that belong to the same sample ontology group as the evaluated epigenome (x-axis). The evaluations are calculated using GECSI-predicted soft chromatin state assignments for 18 chromatin states among 414 fullobs reference epigenomes. Each scatterplot displays the AUROC scores across all fullobs reference epigenomes for one of the chromatin states, where the blue line is the line of best fit of the scatterplot, with the coefficient of determination (R^2) labeled on the top left of each plot.

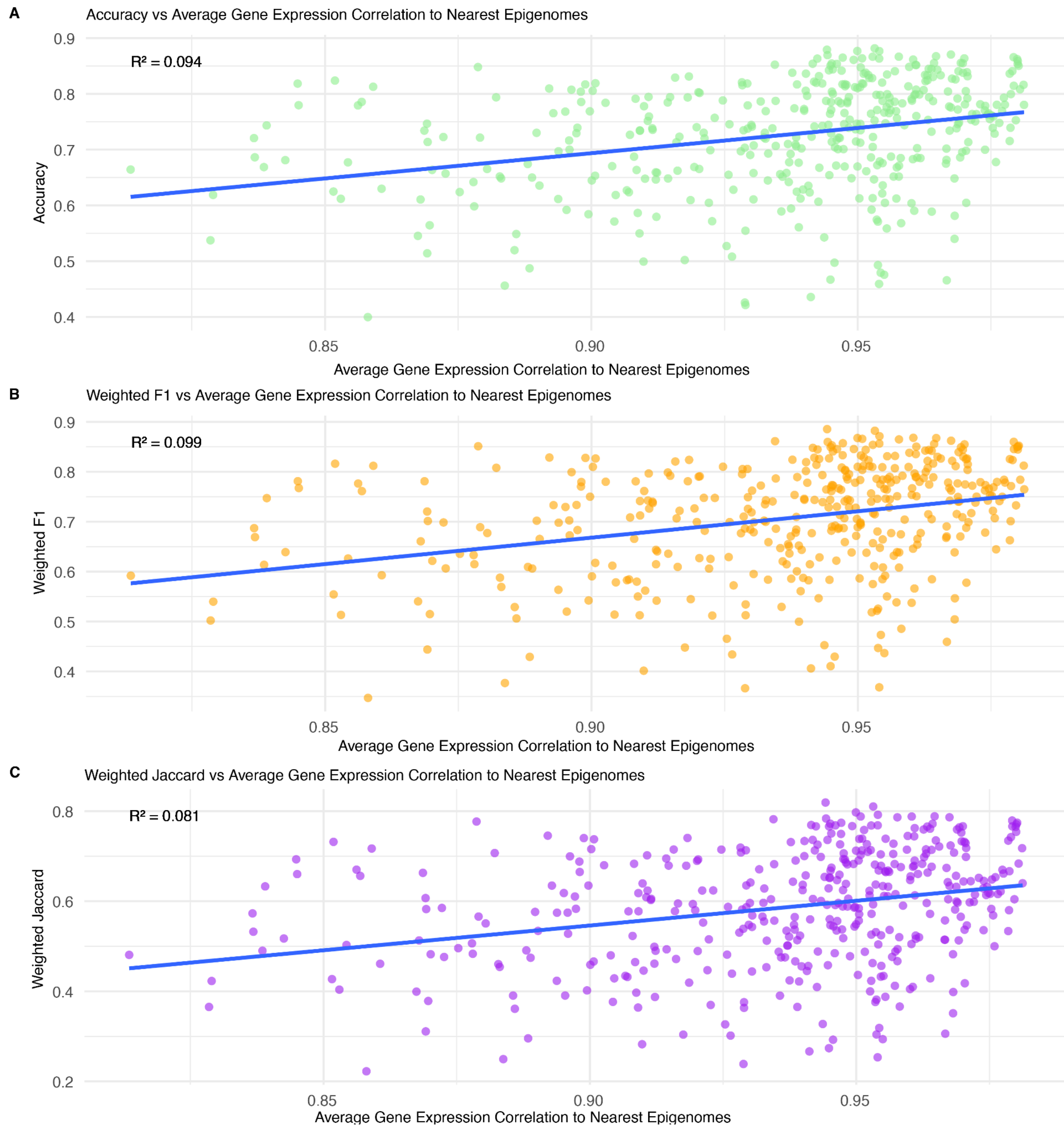

Fig. S13: Evaluation of GECSI's hard assignment predictions agreement with observed assignments as a function of average gene expression correlation to nearest epigenomes. Scatterplots showing cross-validation results for **(A)** accuracy, **(B)** weighted F1 average, and **(C)** weighted Jaccard average (y-axis) with respect to the average gene expression correlation to the target epigenome's 10 nearest epigenomes (x-axis). The evaluations are calculated using GECSI-predicted hard chromatin state assignments for 18 chromatin states among 414 fullobs reference epigenomes. The blue line is the line of best fit of the scatterplot, with the coefficient of determination (R^2) labeled on the top left of each plot.

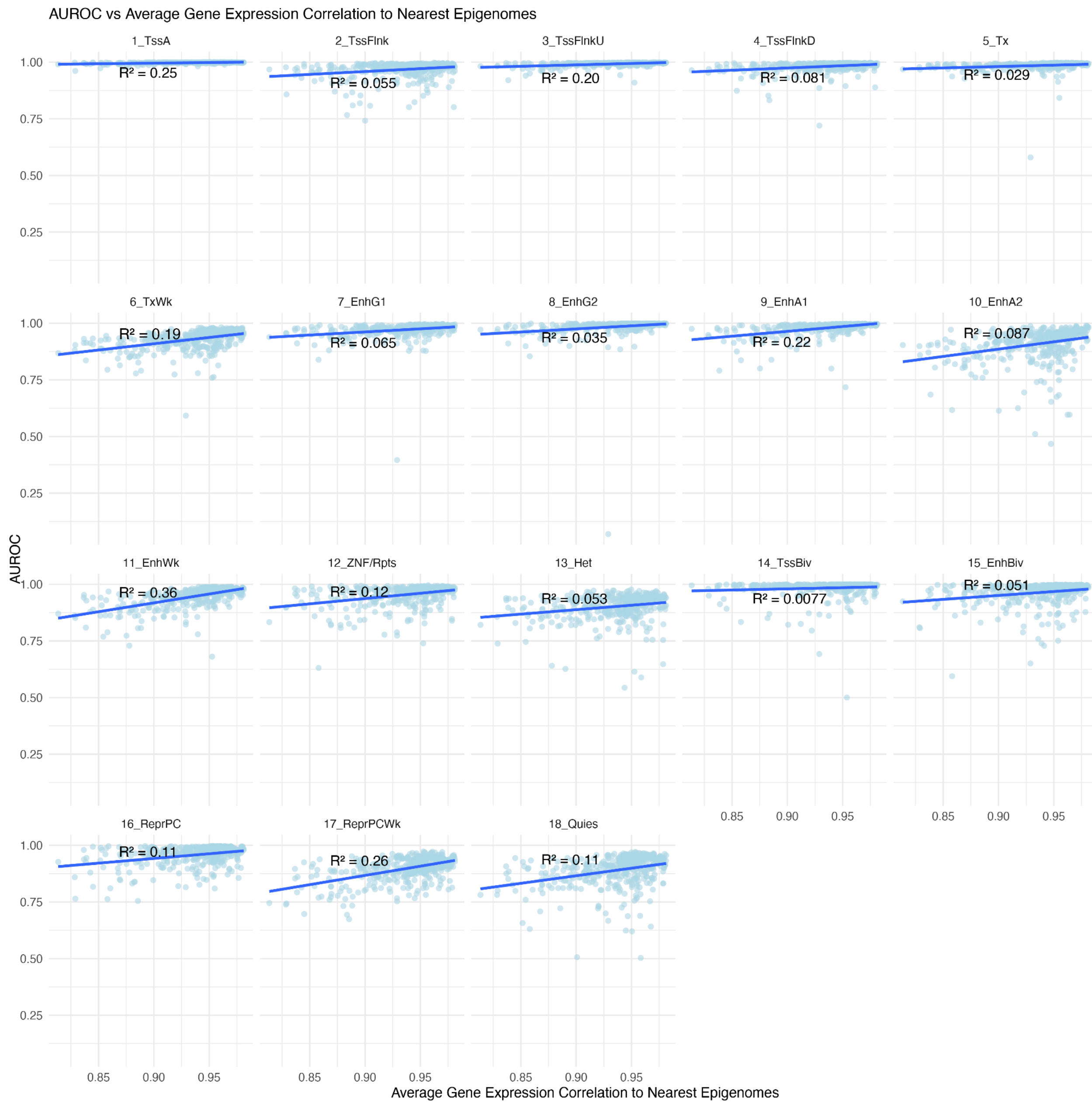

Fig. S14: Evaluation of GECSI's soft assignment predictions' AUROC for predicting observed assignments as a function of average gene expression correlation to nearest epigenomes. Scatterplots showing cross-validation results for AUROC (y-axis) with respect to the average gene expression correlation to the target epigenome's 10 nearest epigenomes (x-axis). The evaluations are calculated using GECSI-predicted soft chromatin state assignments for 18 chromatin states among 414 fullobs reference epigenomes. Each scatterplot displays the AUROC scores across all fullobs reference epigenomes for one of the chromatin states, where the blue line is the line of best fit of the scatterplot, with the coefficient of determination (R^2) labeled on each plot.

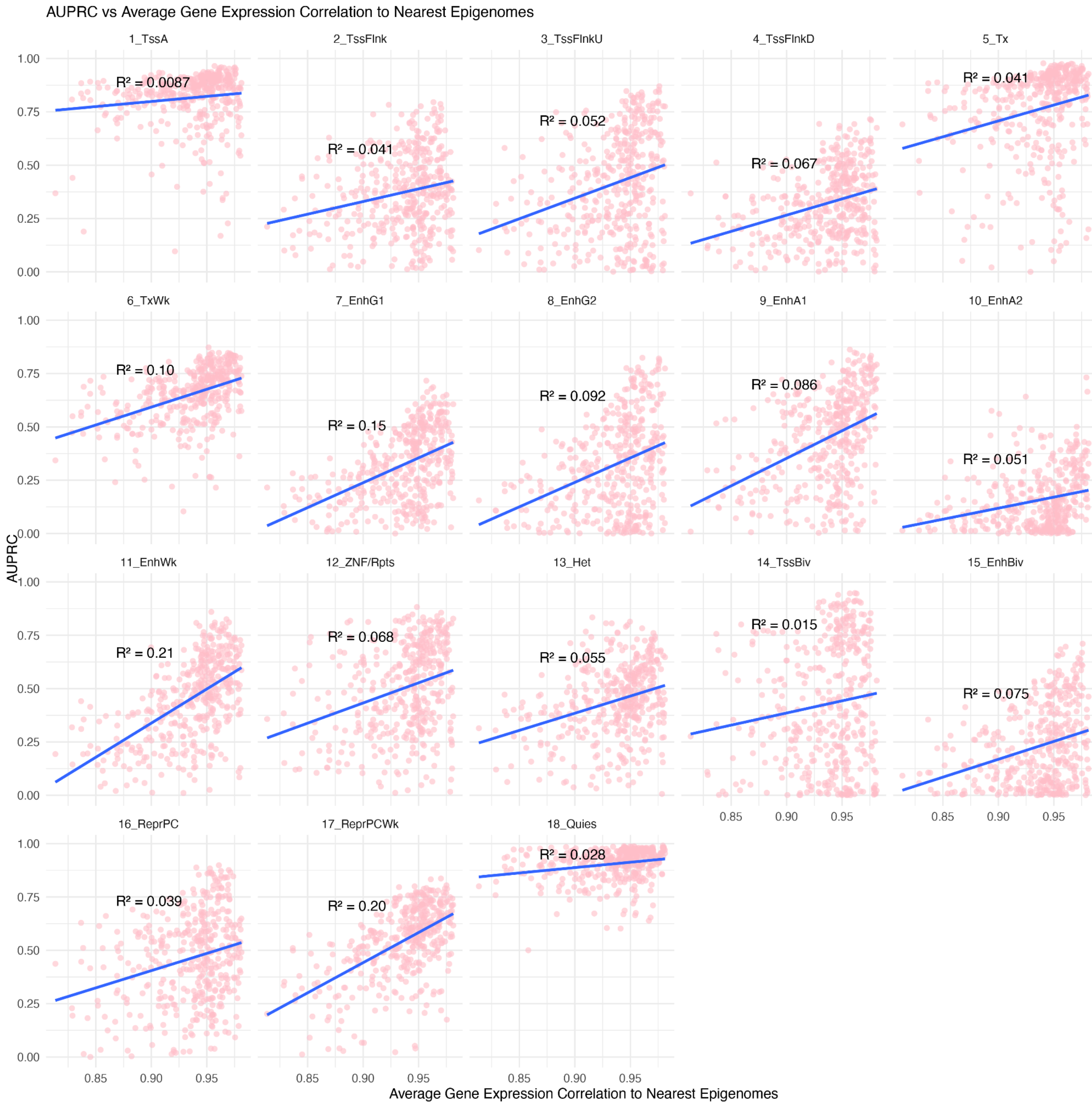

Fig. S15: Evaluation of GECSI's soft assignment predictions' AUPRC for predicting observed assignments as a function of average gene expression correlation to nearest epigenomes. Scatterplots showing cross-validation results for AUPRC (y-axis) with respect to the average gene expression correlation to the target epigenome's 10 nearest epigenomes (x-axis). The evaluations are calculated using GECSI-predicted soft chromatin state assignments for 18 chromatin states among 414 fullobs reference epigenomes. Each scatterplot displays the AUPRC scores across all fullobs reference epigenomes for one of the chromatin states, where the blue line is the line of best fit of the scatterplot, with the coefficient of determination (R^2) labeled on each plot.

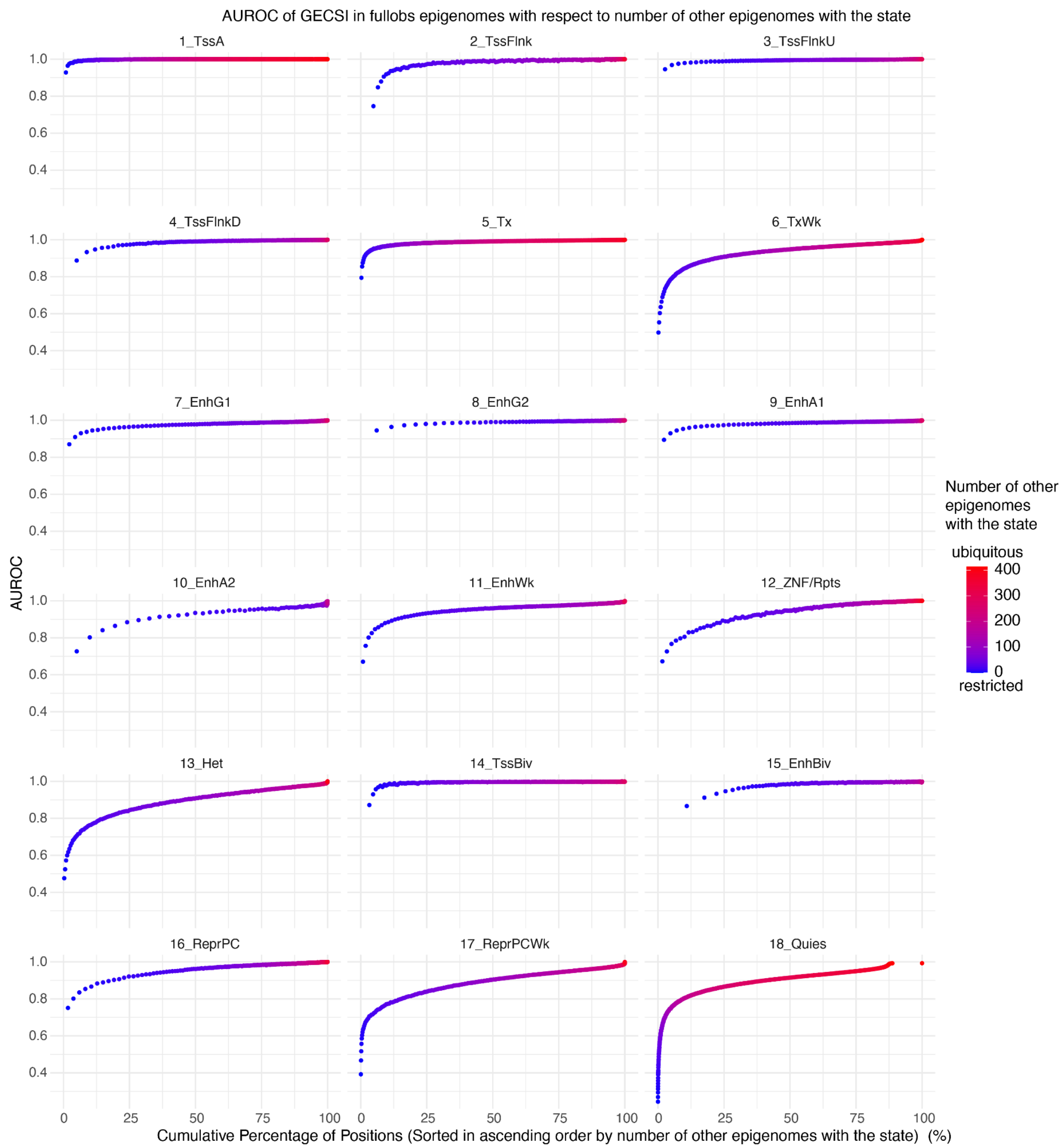

Fig. S16: Evaluation of AUROC for predicting each of the 18 chromatin states as a function of the number of other epigenomes with the state. Scatterplots showing average AUROC of GECSI-predicted soft assignment of a chromatin state (y-axis) as a function of the cumulative percentage of positions sorted in ascending order by the number of other epigenomes with that chromatin state (x-axis), from a cross-validation evaluation among 414 fullobs reference epigenomes (Methods). Each plot displays the values for one out of the 18 chromatin states. Average cross-genome AUROC are computed across all epigenomes. Each dot corresponds to one unique number of other epigenomes with the state and are colored by the number of other epigenomes with the state, with blue being fewer other epigenomes with the state (restricted) and red being more other epigenomes with the state (ubiquitous).

Predicted \ Observed	1_TssA	2_TssFlnk	3_TssFlnkU	4_TssFlnkD	5_Tx	6_TxWk	7_EnhG1	8_EnhG2	9_EnhA1	10_EnhA2	11_EnhWk	12_ZNF/Rpts	13_Het	14_TssBiv	15_EnhBiv	16_ReprPC	17_ReprPCWk	18_Quies
1_TssA	46101	8204	7462	1330	7	743	23	243	301	1303	378	32	8	404	31	38	78	273
2_TssFlnk	4108	12121	1536	3621	9	875	42	80	85	541	952	48	22	1514	291	197	458	757
3_TssFlnkU	3509	1847	18275	3757	8	2369	121	666	3950	1749	2380	10	11	172	45	19	150	715
4_TssFlnkD	493	2623	2946	10724	10	1244	183	256	425	176	3068	21	14	809	255	63	316	659
5_Tx	16	43	31	88	495210	132937	31482	1781	354	1612	1641	4858	617	8	19	225	536	8476
6_TxWk	649	2068	1448	3334	178658	999759	35577	3075	13461	29795	99558	8164	9988	167	663	2328	20006	226858
7_EnhG1	16	62	69	414	5058	3928	17916	5078	864	637	2510	43	6	3	11	11	22	106
8_EnhG2	64	62	362	241	183	723	1788	5296	612	132	330	2	1	1	1	1	2	23
9_EnhA1	515	207	8304	1537	159	8305	2005	4027	56194	11753	21605	58	99	25	127	65	576	2833
10_EnhA2	986	579	1475	488	355	5272	1490	707	5017	9601	3842	41	30	98	101	48	360	1452
11_EnhWk	478	2017	5414	16285	848	48292	9116	2815	34463	9420	159960	329	697	490	2453	1048	7487	30930
12_ZNF/Rpts	17	50	12	38	2043	4551	76	25	73	154	285	95551	45664	53	63	2824	9065	18049
13_Het	7	38	12	31	355	5338	19	4	85	163	640	76026	281462	16	64	2741	29162	128067
14_TssBiv	175	1040	168	987	2	42	2	2	5	45	263	25	6	6736	1368	1260	369	148
15_EnhBiv	17	64	38	159	5	39	11	3	35	14	374	25	10	981	2351	790	256	48
16_ReprPC	64	394	53	186	162	1330	64	15	79	78	990	2015	1531	1876	4436	58421	33903	5751
17_ReprPCWk	141	1041	203	746	972	22475	256	52	921	1723	10993	12378	45521	1174	5636	127327	804309	346950
18_Quies	931	5461	2092	5455	17126	489398	2749	385	12040	20367	116590	67516	577053	1077	4176	54005	930822	7591249

Fig. S17: Confusion matrix between observed and GECSI-predicted chromatin states in the fullobs epigenomes. A confusion matrix showing the average number of positions which are assigned to each combination of GECSI-predicted and observed chromatin states across the whole genome. Each value shows the average number of positions across all positions in the fullobs epigenomes with the corresponding GECSI-predicted (rows) and observed (columns) chromatin states. The color scale is column specific and reflects the individual values normalized by the sum of the values in the column. Darker green reflects a higher proportion while white reflects low proportion. Chromatin states are ordered and colored as previously (Roadmap Epigenomics Consortium et al., 2015). Descriptions that correspond to the chromatin state abbreviations are available in **Additional File 1: Table S1**.

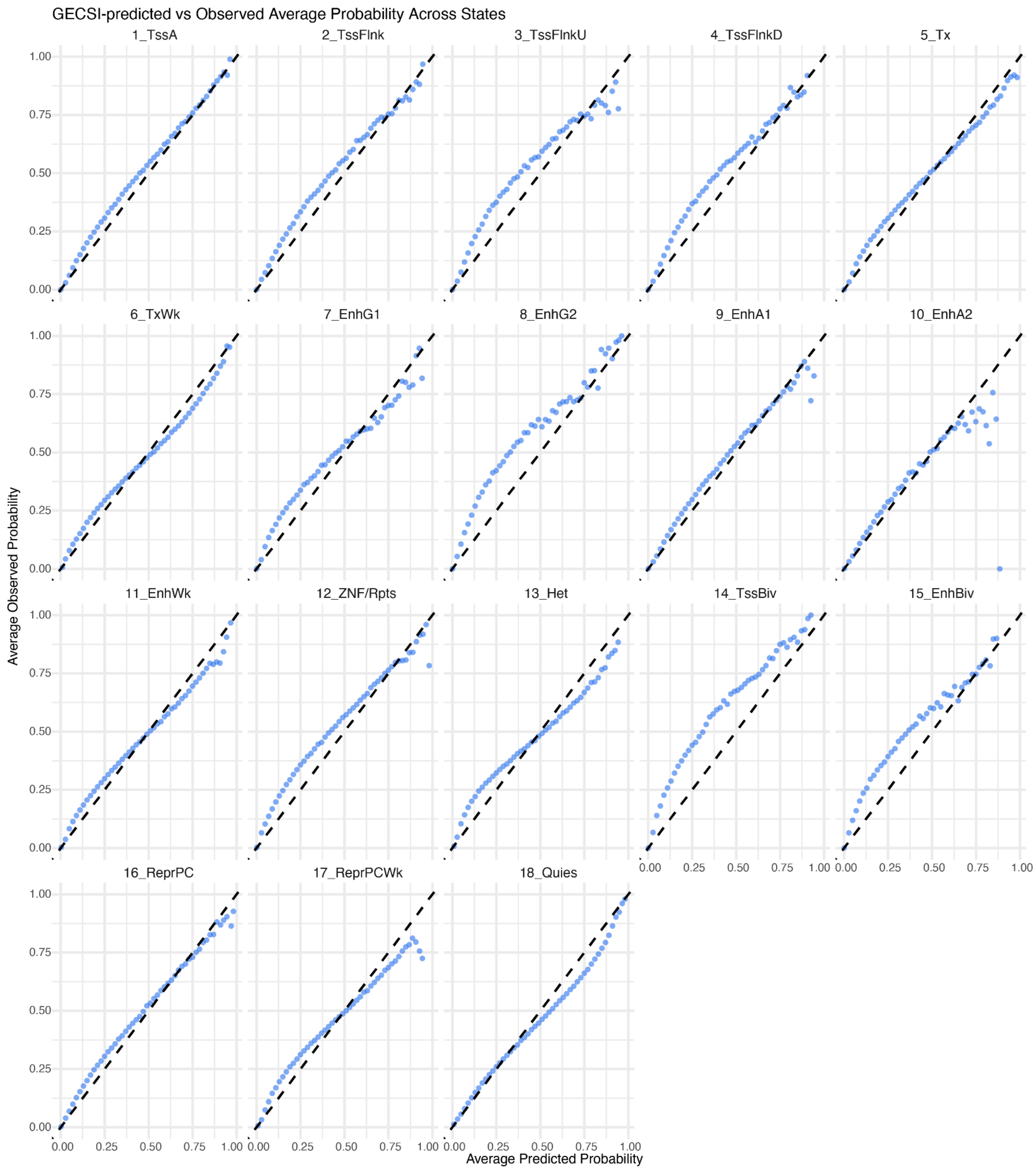

Fig. S18: Calibration analysis of GECSI-predicted probabilities in 18 chromatin states. Scatterplots comparing average GECSI-predicted probabilities for a chromatin state (x-axis) to the average observed frequency of the state (y-axis) from a cross-validation evaluation among 414 fullobs reference epigenomes. Each plot displays the values for one out of the 18 chromatin states. For each chromatin state and epigenome, genomic positions were grouped based on the predicted probability of the state in the epigenome and divided into 50 equal sub-intervals of the interval [0,1]. Average predicted probabilities and average observed probabilities are computed across all positions in each group and all epigenomes. The diagonal black dashed line marks the y=x line, representing perfect calibration.

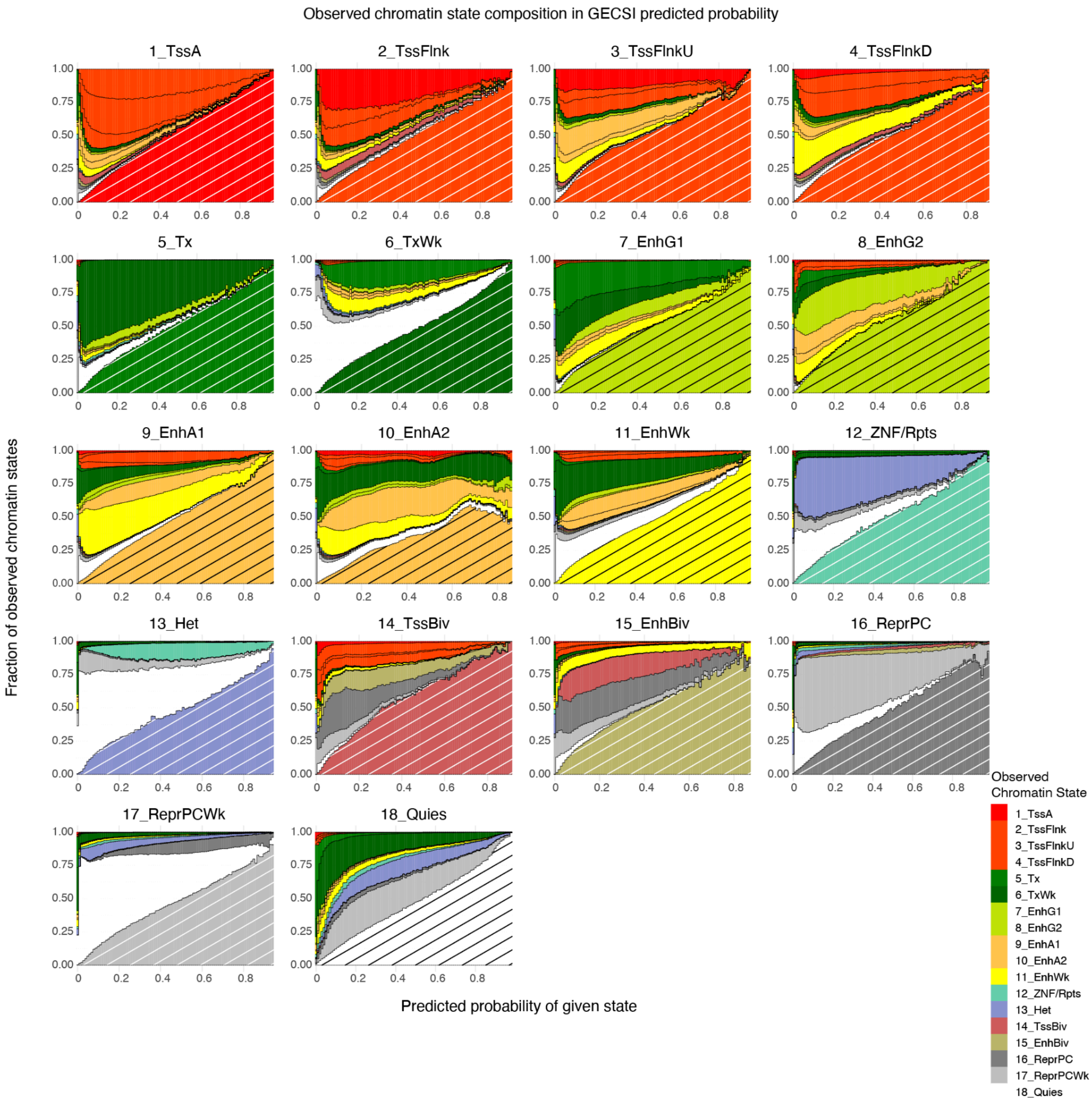

Fig. S19: Visualization of observed chromatin state composition with respect to GECSI-predicted probabilities for the 18 chromatin states in the fullobs epigenomes. Each plot shows the distribution of the observed chromatin states conditioned on GECSI's predicted probabilities of a chromatin state, which are grouped into 100 bins based on the predicted probability of the given state shown on the x-axis. Only bins that had more than or equal to 100 positions assigned to it are displayed and the rest are filtered out and omitted as they only occur at the ends of the available probability ranges. The bars that represent the fractions of a chromatin state are colored by the color-coding of that state. For each plot, the color bar corresponding to the given state is located at the bottom of each bar with diagonal shading overlaid for better visualization. The remaining chromatin states are stacked above it, following the standard order of the chromatin states.

Observed

State	CpG sites	Exons	Genes	TES	TSS	TSS 2kb
1_TssA	73.8	10.3	1.8	1.9	71.0	12.5
2_TssFlnk	49.1	6.4	1.5	1.9	40.2	10.4
3_TssFlnkU	15.8	4.5	1.7	2.8	10.8	8.7
4_TssFlnkD	15.6	4.5	1.7	3.0	9.7	9.3
5_Tx	0.8	5.2	2.3	6.8	2.4	3.7
6_TxWk	0.3	1.8	1.9	1.9	0.9	1.8
7_EnhG1	1.0	5.6	2.2	8.5	3.1	4.1
8_EnhG2	1.8	6.9	2.2	11.4	4.4	5.3
9_EnhA1	0.2	1.3	1.5	1.3	1.4	2.1
10_EnhA2	0.9	1.3	1.4	1.3	1.7	2.2
11_EnhWk	0.5	1.2	1.5	1.1	1.2	2.2
12_ZNF/Rpts	0.8	1.6	0.9	1.2	0.4	0.6
13_Het	0.2	0.2	0.6	0.2	0.1	0.2
14_TssBiv	88.1	8.4	1.5	2.1	26.0	10.1
15_EnhBiv	34.3	4.4	1.3	2.5	8.2	6.3
16_ReprPC	4.7	1.7	1.0	1.3	1.8	2.8
17_ReprPCWk	0.4	0.7	0.9	0.6	0.5	0.8
18_Quies	0.2	0.4	0.8	0.4	0.2	0.4

GECSI predicted

State	CpG sites	Exons	Genes	TES	TSS	TSS 2kb
1_TssA	74.7	10.3	1.8	1.9	70.7	12.7
2_TssFlnk	67.8	7.3	1.6	1.9	46.8	11.4
3_TssFlnkU	11.6	4.6	1.7	3.0	9.5	9.5
4_TssFlnkD	18.2	5.1	1.8	3.2	10.7	11.0
5_Tx	0.8	5.5	2.3	7.4	2.6	3.9
6_TxWk	0.4	1.9	2.0	2.0	0.9	1.9
7_EnhG1	1.1	6.6	2.3	10.9	3.8	4.8
8_EnhG2	1.9	8.0	2.2	13.7	5.1	6.0
9_EnhA1	0.2	1.4	1.5	1.4	1.4	2.2
10_EnhA2	2.5	1.7	1.4	1.8	3.1	3.1
11_EnhWk	0.6	1.3	1.5	1.3	1.4	2.6
12_ZNF/Rpts	0.9	2.4	1.1	1.9	0.6	0.8
13_Het	0.3	0.3	0.5	0.2	0.1	0.2
14_TssBiv	101.5	9.2	1.6	2.2	29.7	10.8
15_EnhBiv	64.4	6.2	1.4	2.9	12.9	8.2
16_ReprPC	10.9	2.5	1.1	1.6	3.1	4.5
17_ReprPCWk	0.6	0.8	0.9	0.7	0.6	1.0
18_Quies	0.2	0.4	0.8	0.4	0.3	0.4

Fig. S20: External genomic annotation and CpG island enrichments in different GECSI-predicted chromatin states across the fullobs epigenomes. Heatmaps showing chromatin-state-specific enrichments of different external categories based on observed chromatin state annotations (left) and GECSI's predictions (right). Each column corresponds to an external category, specifically CpG islands, exons, gene bodies (Genes), transcription end sites (TES), transcription start sites (TSS) and 2kb around TSS (TSS 2kb). Each row represents a chromatin state as shown in the first column of color labels. Each grid represents a median enrichment value of the corresponding chromatin state and external category across all reference epigenomes. The coloring scheme is based on enrichment values normalized for each column separately, where yellow represents the highest enrichment in each column and dark blue represents the lowest enrichment in the column. Specifically, yellow represents higher normalized enrichments and navy blue represents lower normalized enrichments.

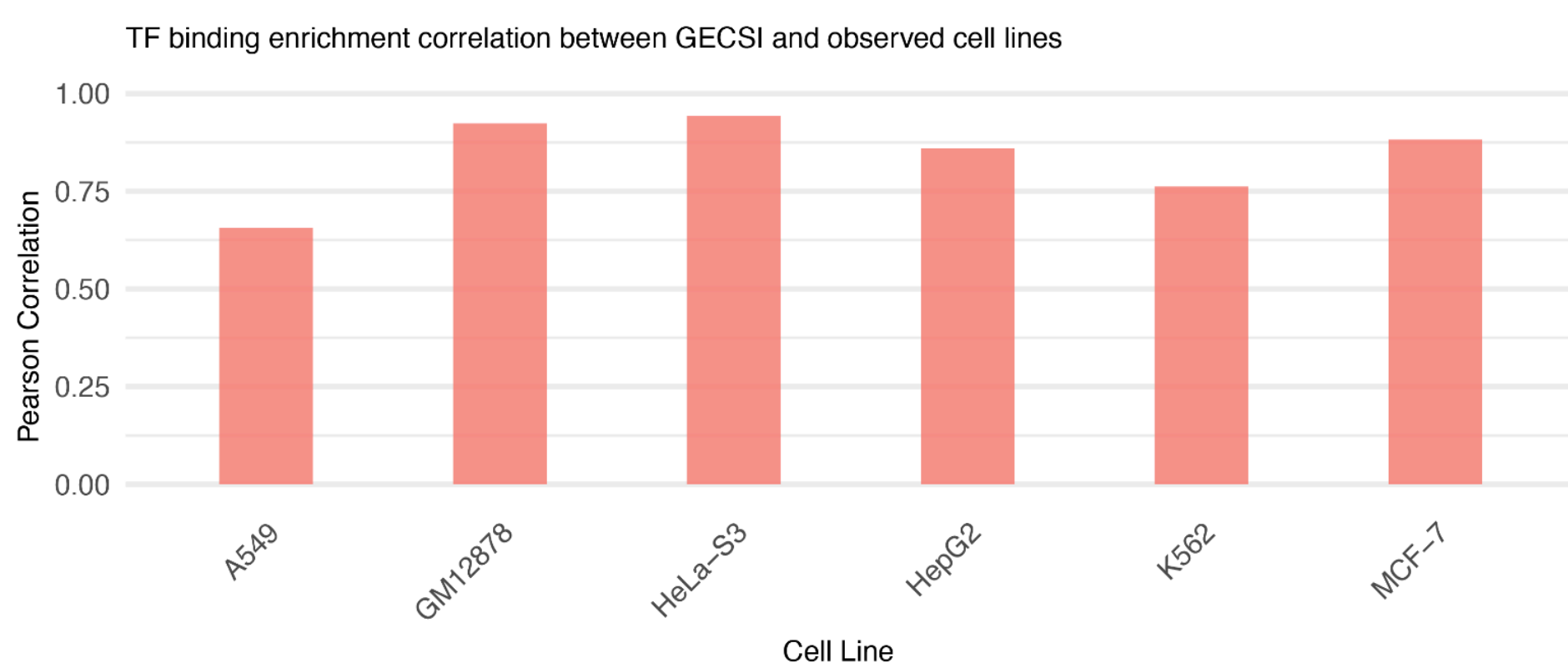

Fig. S21: TF binding enrichment correlation bar plot. Barplot showing for each cell line the correlation between GECSI's predicted chromatin states and observed chromatin states, based on their fold enrichments computed using peak calls from TF ChIP-seq experiments. Each bar represents a Pearson correlation score for a cell line, where the correlation is calculated between the fold enrichments computed in observed chromatin states and the fold enrichments computed in GECSI-predicted chromatin states. The correlation values are based on 58 experiments for A549, 138 for GM12878, 47 for HeLa-S3, 629 for HepG2, 520 for K562, and 108 for MCF-7.

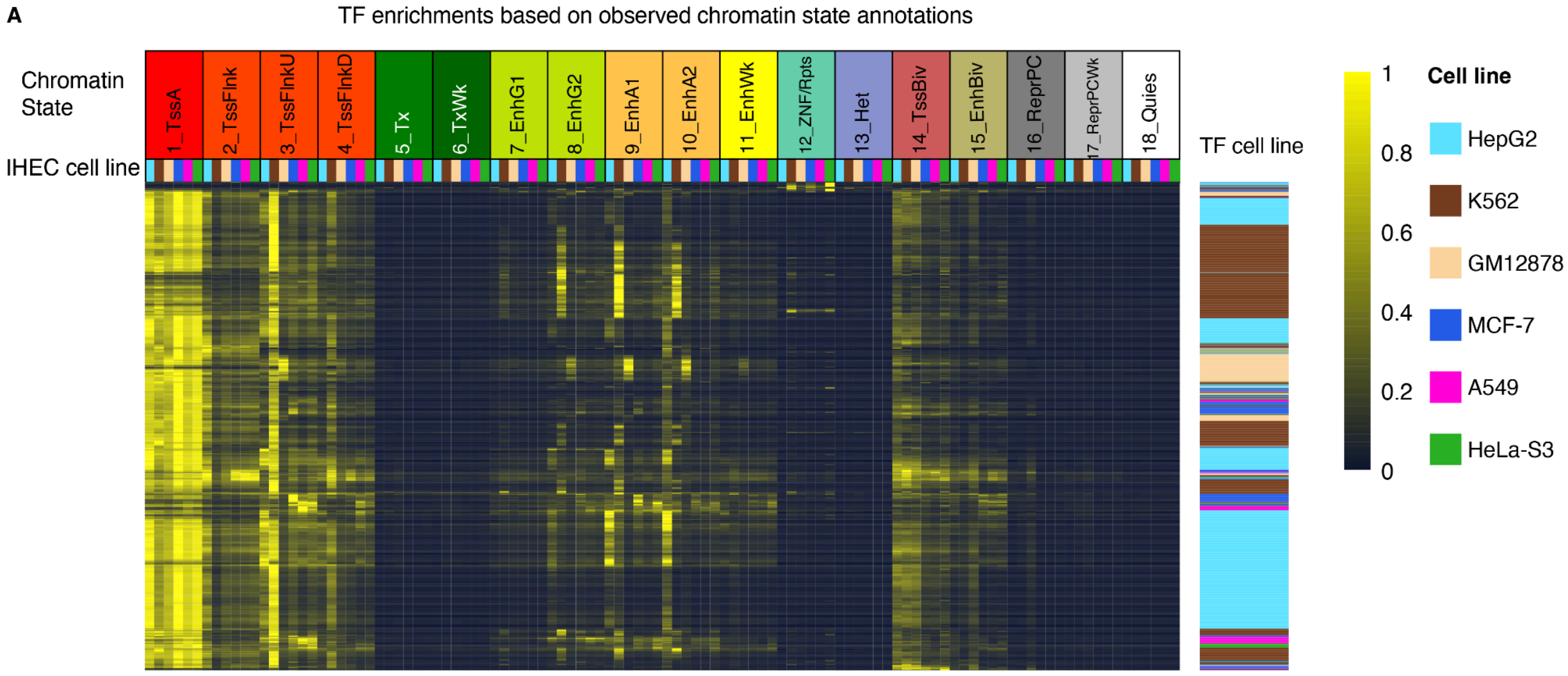

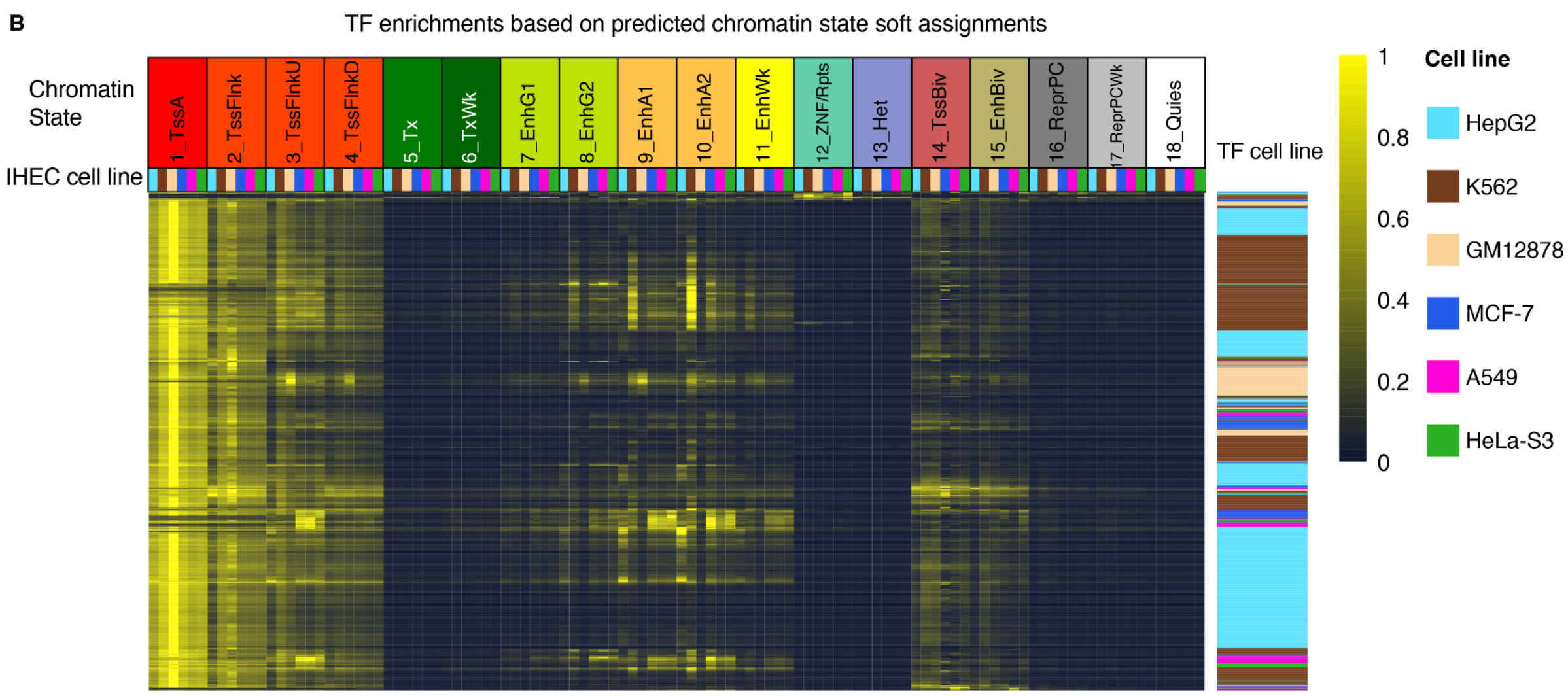

Fig. S22: Transcription factor (TF) binding enrichments in observed chromatin annotations and GECSI-predicted chromatin state soft assignments across six cell lines. (A) TF binding enrichment heatmap with fold enrichments calculated using peak calls from TF ChIP-seq experiments and observed chromatin state hard assignments. The corresponding TF binding enrichment heatmap with fold enrichments calculated using GECSI-predicted chromatin state hard assignments can be found in Fig. 5A. **(B)** TF binding enrichment heatmap with the fold enrichments calculated using peak calls from TF ChIP-seq experiments and GECSI-predicted chromatin state soft assignments with low probabilities (<0.05) truncated to zero and renormalized (Methods). For both (A) and (B), each row corresponds to a TF ChIP-seq experiment in a given cell line. Each column corresponds to a chromatin state-cell line combination. The entries in the heatmap are fold enrichments normalized between 0 and 1 for each row separately, where the maximum is scaled to 1 and the minimum to 0. The top right color scale shows the scaled enrichments with yellow representing higher enrichments and blue representing lower enrichments. The row ordering of TF ChIP-seq experiments for (A) and (B) were obtained by applying a traveling salesman problem solver to the pairwise correlations of the chromatin state enrichments values in (A)

(Methods) and the same ordering was then applied in (B). The columns are ordered first by chromatin states and then by individual cell lines using the following order: HepG2, K562, GM12878, MCF-7, A549, and HeLa-S3. The top row of labels corresponds to chromatin states and the second row of color labels corresponds to cell lines. The rows are color-coded by the cell line of the TF ChIP-seq experiment, with the same color-coding as the column labels.

State	Pearson
1_TssA	0.443
2_TssFlnk	0.519
3_TssFlnkU	0.581
4_TssFlnkD	0.498
5_Tx	0.546
6_TxWk	0.569
7_EnhG1	0.665
8_EnhG2	0.662
9_EnhA1	0.773
10_EnhA2	0.728
11_EnhWk	0.771
12_ZNF/Rpts	0.496
13_Het	0.507
14_TssBiv	0.580
15_EnhBiv	0.487
16_ReprPC	0.621
17_ReprPCWk	0.658
18_Quies	0.745

Fig. S23: Pearson correlation between pairwise sample correlation values derived from observed and GECSI-predicted chromatin states in the fullobs epigenomes. The figure shows for each chromatin state the Pearson correlation between pairwise sample correlations computed based on observed and GECSI's predicted chromatin states. Pairwise sample correlation scores were calculated in the fullobs epigenomes based on the binary presence or absence of the state.

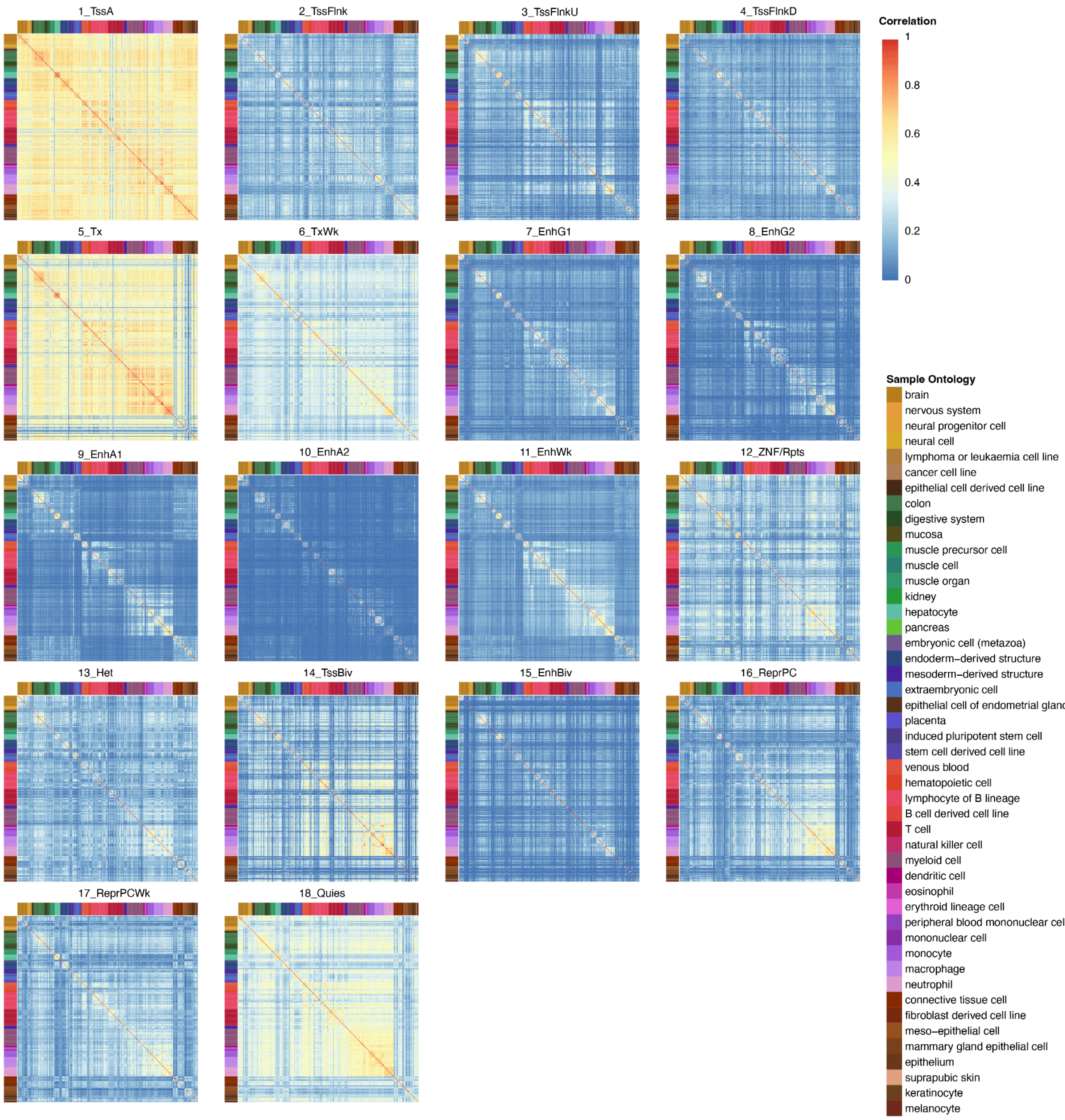

Fig. S24: Pairwise sample correlation generated from observed chromatin state annotations in 18 chromatin states in the fullobs samples. Heatmaps showing for each of the 18 chromatin states the pairwise correlation calculated using the presence or absence of the observed chromatin state among 414 fullobs IHEC epigenomes. In each heatmap, each row and column represents an epigenome, and each entry represents a Pearson correlation value between the epigenome of the row and the epigenome of the column. Only epigenomes with a corresponding chromatin state assignment are shown. The color of the entries follows the color scale on the top right of the figure. Correlation results are ordered based on the provided sample ordering from IHEC (International Human Epigenome Consortium et al., 2026), and color annotation of each sample is based on the sample intermediate ontology in the IHEC metadata using the IHEC coloring.

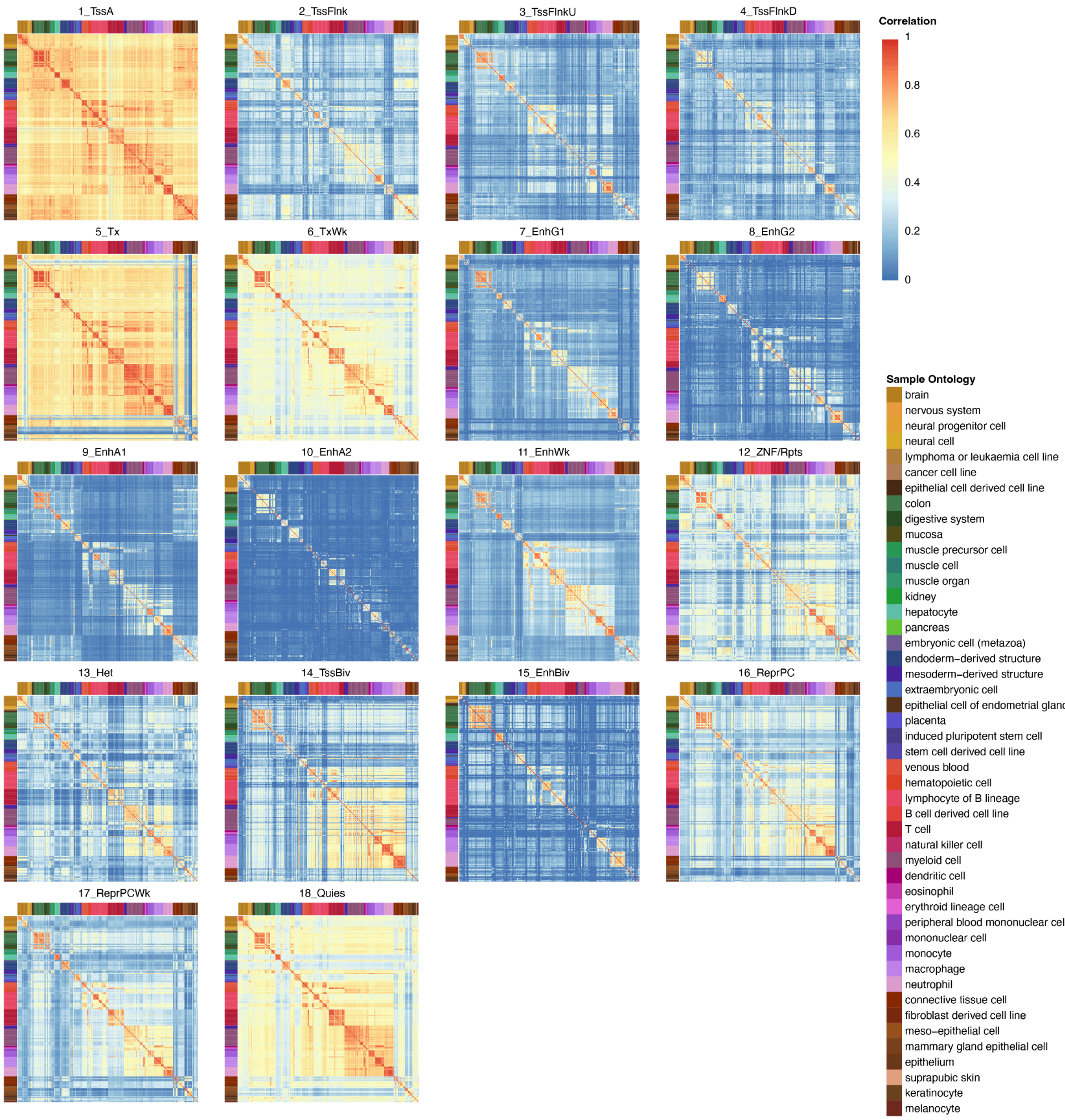

Fig. S25: Pairwise sample correlation generated from GECSI-predicted chromatin state annotations in 18 chromatin states in the fullobs samples. Heatmaps showing for each of the 18 chromatin states the pairwise correlation calculated using the presence or absence of the GECSI-predicted chromatin state among 414 fullobs IHEC epigenomes. In each heatmap, each row and column represents an epigenome, and each entry represents a Pearson correlation value between the epigenome of the row and the epigenome of the column. Only epigenomes with a corresponding chromatin state assignment are shown. The color of the entries follows the color scale on the top right of the figure. Correlation results are ordered based on the provided sample ordering from IHEC (International Human Epigenome Consortium et al., 2026), and color annotation of each sample is based on the sample intermediate ontology in the IHEC metadata using the IHEC coloring.

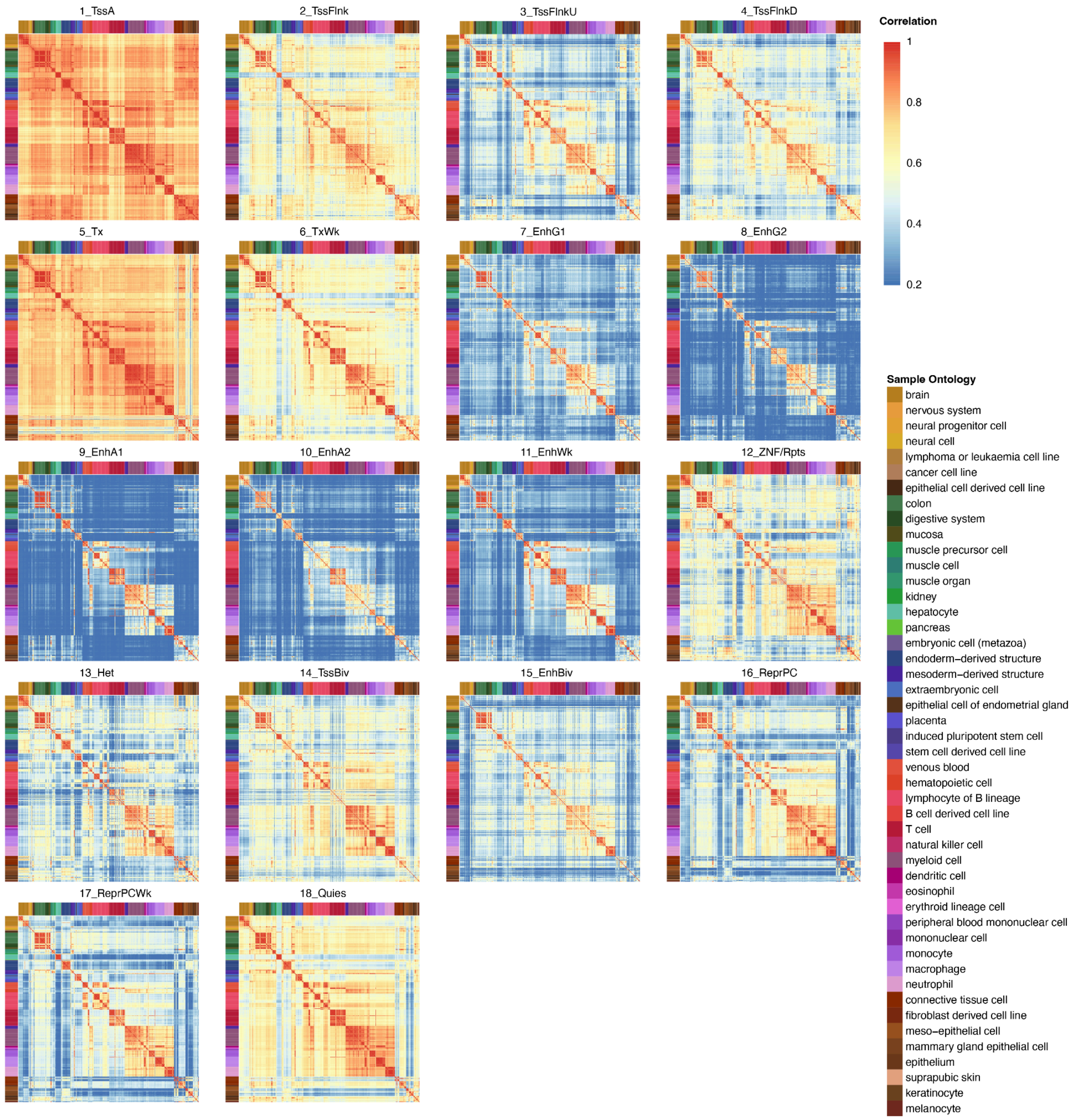

Fig. S26: Pairwise sample correlation generated from GECSI-predicted chromatin state soft assignments in 18 chromatin states in the fullobs epigenomes. Heatmaps showing for each of the 18 chromatin states the pairwise correlation calculated using the soft assignments of the GECSI-predicted chromatin state among the 414 fullobs IHEC epigenomes. In each heatmap, each row and column represents an epigenome, and each entry represents a Pearson correlation value between the epigenome of the row and the epigenome of the column. The color of the entries follows the color scale on the top right of the figure (0.2–1.0), with values below 0.2 mapped to the color for 0.2. Correlation results are ordered based on the provided sample ordering from IHEC (International Human Epigenome Consortium et al., 2026), and color annotation of each sample is based on the sample intermediate ontology in the IHEC metadata using the IHEC coloring.

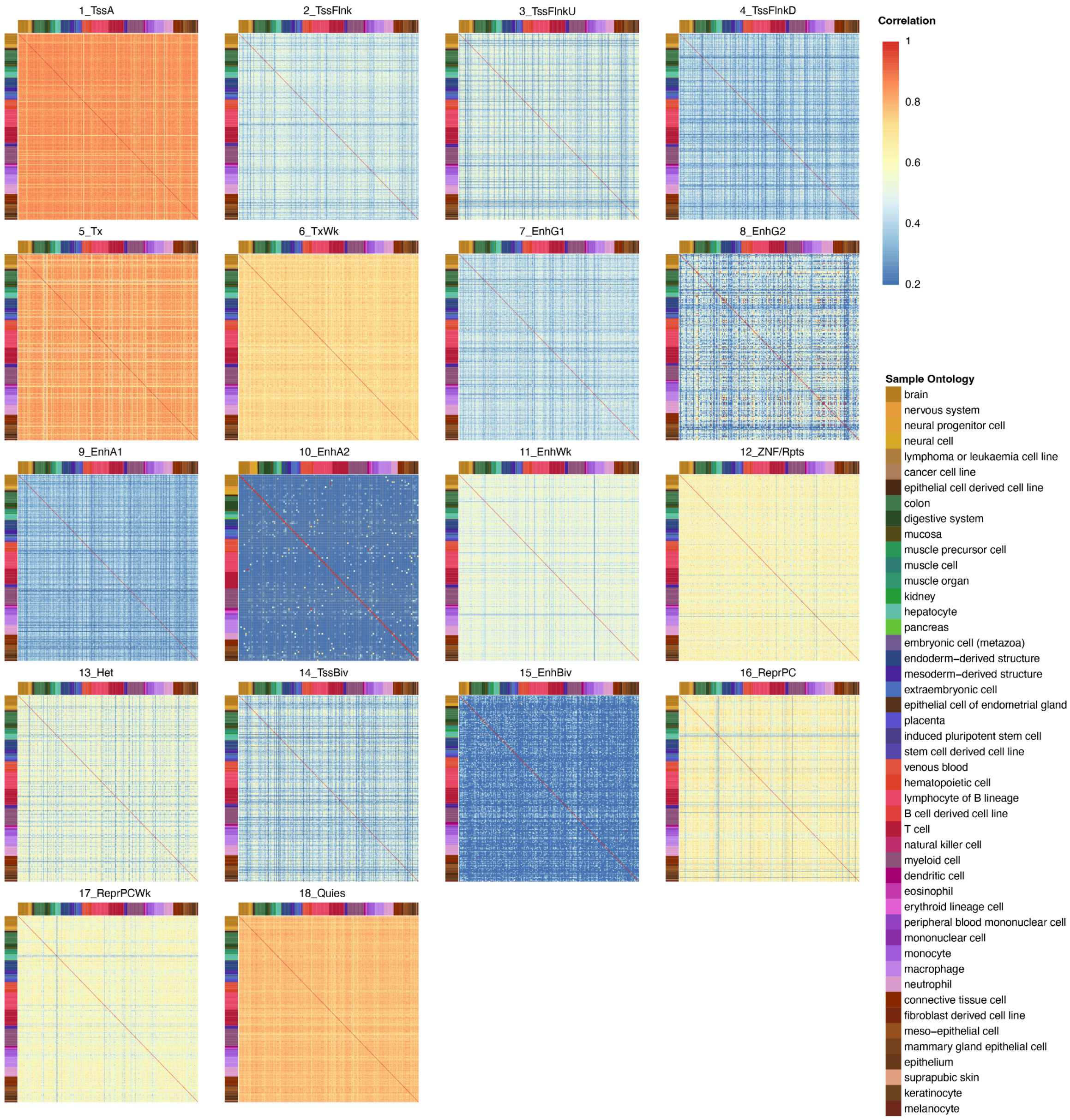

Fig. S27: Pairwise sample correlation generated from permutation based experimentally predicted chromatin state annotations in 18 chromatin states in the fullobs samples. Heatmaps showing for each of the 18 chromatin states the pairwise correlation calculated using the presence or absence of the permutation based experimentally predicted chromatin state among 414 fullobs IHEC epigenomes (Methods). In each heatmap, each row and column represents an epigenome, and each entry represents a Pearson correlation value between the epigenome of the row and the epigenome of the column. Only epigenomes with a corresponding chromatin state assignment are shown. The color of the entries follows the color scale on the top right of the figure. Correlation results are ordered based on the provided sample ordering from IHEC (International Human Epigenome Consortium et al., 2026), and color annotation of each sample is based on the sample intermediate ontology in the IHEC metadata using the IHEC coloring as indicated in the bottom right color legend.

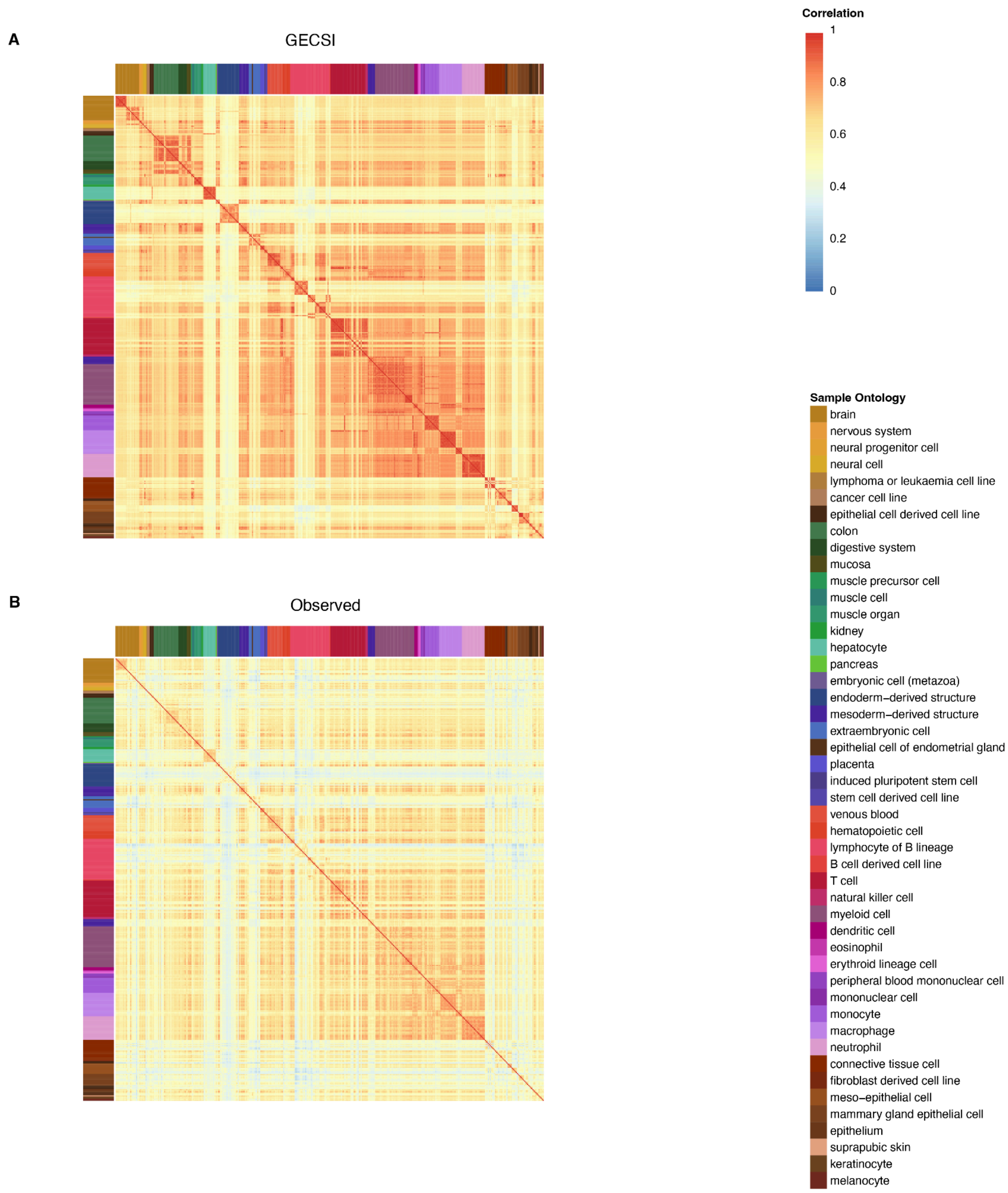

Fig. S28: Pairwise sample agreements generated from GECSI predicted and observed chromatin state annotations. Heatmaps showing the pairwise agreements calculated using **(A)** GECSI-predicted and **(B)** observed chromatin states among the 414 fullobs IHEC epigenomes. Each row and column represents an epigenome, and each value represents an agreement score between the epigenome of the row and the epigenome of the column. The color of the entries follows the color scale on the top right of the figure. Correlation results are ordered based on the provided sample ordering from IHEC (International Human Epigenome Consortium et al., 2026), and color annotation of each sample is based on sample intermediate ontology in the IHEC metadata using the color codes provided by IHEC.

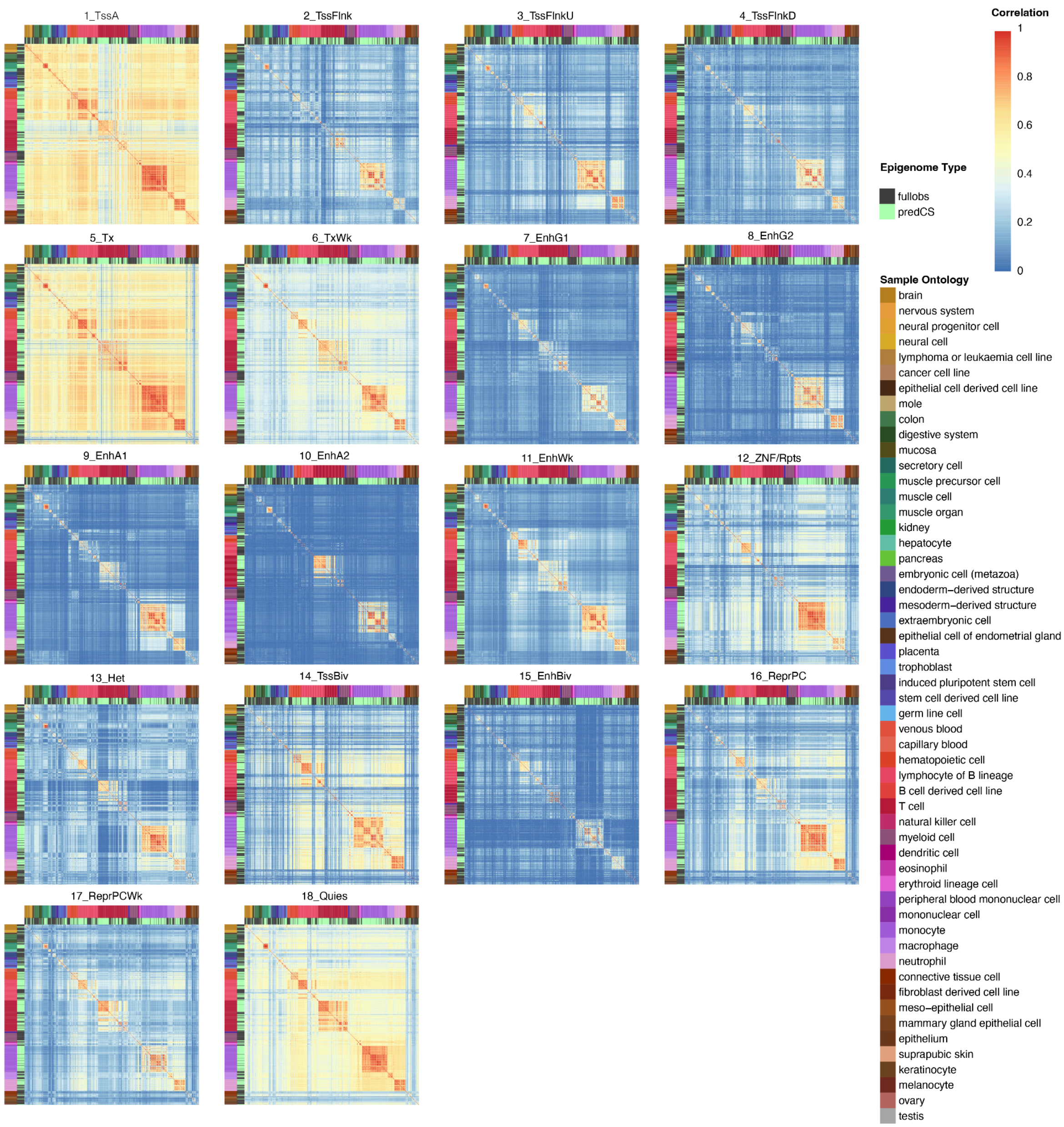

Fig. S29: Pairwise sample correlation generated from observed + GECSI imputed chromatin states in 18 chromatin states in the fullobs and predCS epigenomes. Heatmaps showing for each of the 18 chromatin states the pairwise correlation calculated using the binary presence or absence of the observed chromatin states among the 414 fullobs IHEC epigenomes and GECSI-predicted chromatin states among the 449 predCS epigenomes. In each heatmap, each row and column represents an epigenome, and each entry represents a Pearson correlation between the epigenome of the row and the epigenome of the column. Only epigenomes with a corresponding chromatin state assignment are shown. The color of the entries follows the color scale shown on the top right. Correlation results are ordered based on the provided sample ordering from IHEC (International Human Epigenome Consortium et al., 2026). The outer multiple-color annotation of epigenomes is based on sample intermediate ontology in the IHEC metadata using the IHEC coloring, while the inner binary color annotation is based on whether the chromatin state is observed (in the fullobs epigenomes) or GECSI-predicted (in the predCS epigenomes).

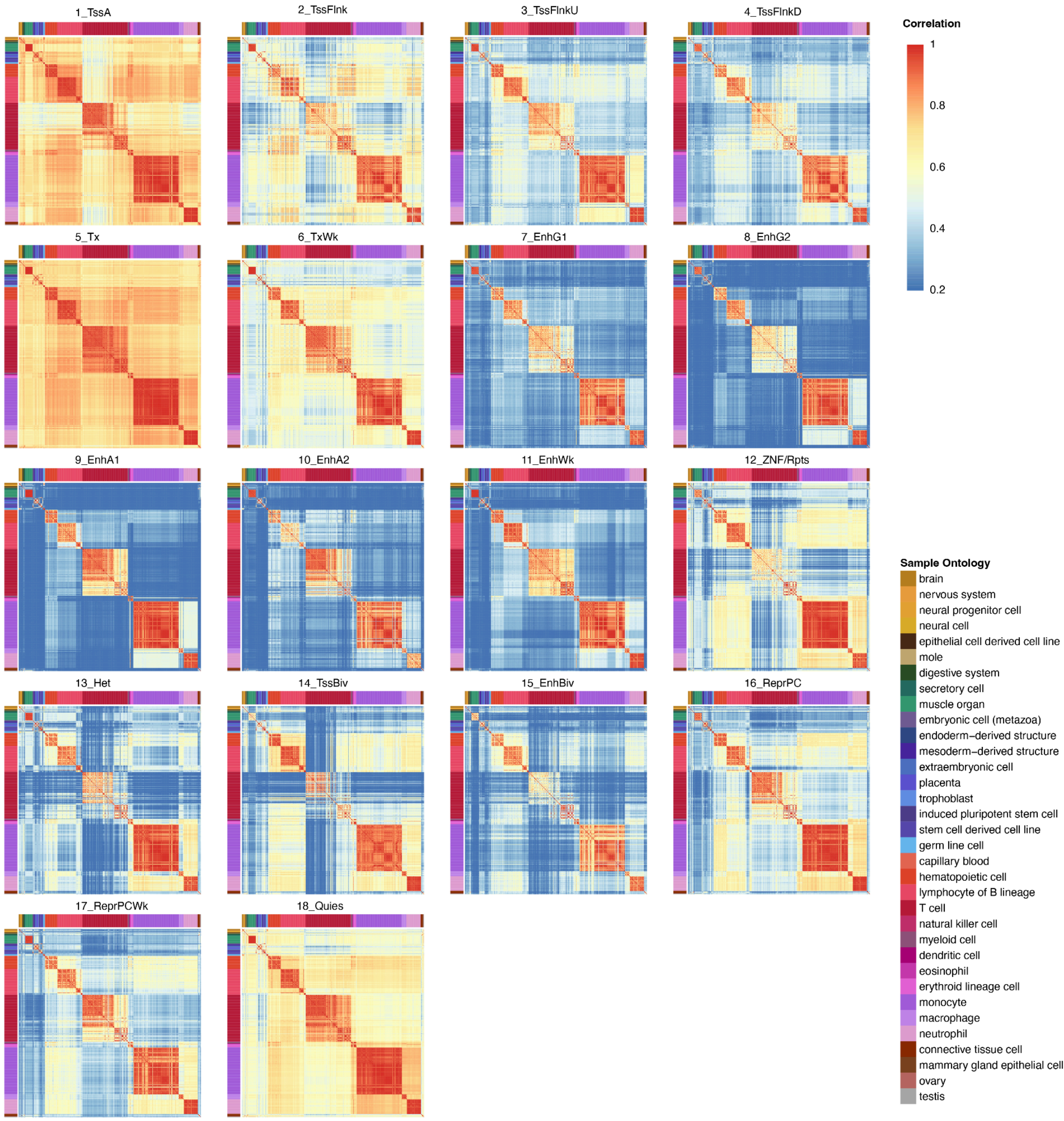

Fig. S30: Pairwise sample correlation generated from GECSI-imputed chromatin state soft assignments in 18 chromatin states in the predCS epigenomes. Heatmaps showing for each of the 18 chromatin states the pairwise correlation calculated using the soft assignments of the chromatin states among 449 GECSI-predicted chromatin states from predCS epigenomes. In each heatmap, each row and column represents an epigenome, and each entry represents a Pearson correlation between the epigenome of the row and the epigenome of the column. The color of the entries follows the color scale on the top right of the figure (0.2–1.0), with values below 0.2 mapped to the color for 0.2. Correlation results are ordered based on the provided sample ordering of IHEC (International Human Epigenome Consortium et al., 2026), and color annotation of each sample is based on the sample intermediate ontology in the IHEC metadata using the IHEC coloring.
